# Phylogenomic insights into the genus *Tulipa* (Liliaceae): Taxonomy, evolution, and biogeography

**DOI:** 10.1002/ajb2.70232

**Published:** 2026-08-04

**Authors:** Brett Wilson, Maarten J. M. Christenhusz, Nathanael Walker‐Hale, Natalya Beshko, Mariyo Boboev, Colin Clubbe, Davron Dekhkonov, Aibek Dolotbakov, Mehmet Firat, Thomas Freeth, Sjaak de Groot, Avni Hajdari, Georgy A. Lazkov, Ormon Sultangaziev, Komiljon S. Tojibaev, Ben J. M. Zonneveld, Kaiyrkul T. Shalpykov, Samuel F. Brockington

**Affiliations:** ^1^ Department of Plant Sciences University of Cambridge Cambridge UK; ^2^ Royal Botanic Gardens Kew Richmond Surrey UK; ^3^ School of Biological Sciences University of Western Australia Perth Australia; ^4^ Institute of Botany Uzbekistan Academy of Sciences Durmon yuli str. 32 Tashkent 100125 Uzbekistan; ^5^ Kulob Botanical Garden Khatlon Scientific Center of the National Academy of Sciences of Tajikistan Kulob 735360 Tajikistan; ^6^ Namangan State University Boburshox str., 161 Namangan 160107 Uzbekistan; ^7^ Institute of Chemistry and Phytotechnology National Academy of Sciences of the Kyrgyz Republic Bishkek Kyrgyzstan; ^8^ Faculty of Education, Department of Biology Van Yüzüncü Yıl University Van TR‐65080 Turkey; ^9^ Beeklaan 19 Lisse (de Zilk) 2191AA The Netherlands; ^10^ Department of Biology, Faculty of Mathematics and Natural Science University of Prishtina “Hasan Prishtina” Prishtinë Kosovo; ^11^ Institute of Biology National Academy of Sciences of the Kyrgyz Republic Bishkek Kyrgyz Republic; ^12^ Research Center for Ecology and Environment of Central Asia (Bishkek) Bishkek Kyrgyz Republic; ^13^ Fauna & Flora International Bishkek Kyrgyz Republic; ^14^ Naturalis Biodiversity Center, Tropical Botany Section Darwinweg 2 Leiden 2333CR The Netherlands

**Keywords:** biogeography, bulbous monocots, conservation, geophytes, phylogenetics, plastome, taxonomy, tulips, Биогеография, однодольные луковичные, сохранение, геофиты, филогенетика, пластом, таксономия, тюльпаны

## Abstract

**Premise:**

Tulips are one of the best‐known geophytes, but their taxonomy remains convoluted and their evolutionary history poorly understood. Here, we used plastid genomes to identify some issues with current classification, understand the diversification history of the genus, and identify potential species linked to historical cultivation.

**Methods:**

We gathered a large number of tulip specimens from living collections, herbaria, and online sources and through extensive fieldwork to infer maximum likelihood phylogenies of the plastid genomes of *Tulipa* (Liliaceae), representing ~86% of accepted species alongside a range of occasionally accepted synonyms. We used BEAST and secondary calibration points to date the tree, before assessing the biogeographical history of the genus using BioGeoBEARS.

**Results:**

Based on our results, we described a fifth subgenus, *Eduardoregelia*, and identified a range of species where our results do not align with current taxonomy. Our data suggests the genus diverged from a clade containing *Amana* and *Erythronium* around 32.5 million years ago (Mya), with the most recent common ancestor existing around 22.8 Mya in the broader Central Asia region, a period that saw rapid uplift of mountain ranges and associated aridification in the region. The genus primarily diversified in Central Asia with rapid radiations within the last 10 My, possibly as a response to further orogenesis. Several migrations outside of the ancestral region have occurred, primarily via the steppes of Kazakhstan and Russia and along the Caucasus and Iranian and Anatolian Mountains to the eastern Mediterranean region. We also provide evidence that the maternal lineage of the garden tulip involved several species including *T. scardica* and *T. suaveolens*.

**Conclusions:**

Many tulip species present limited diagnostic characters, making their taxonomy difficult, compounded by their long use in horticulture and widespread hybridization, especially in gardens. We provide an up‐to‐date phylogenetic framework for this genus that may help resolve many taxonomic issues in the future, while highlighting further work to be done. We reinforce the importance of Central Asia in the evolutionary history of this genus and provide an important foundation for further study of *Tulipa* and more effective conservation of wild tulip species.

## АННОТАЦИЯ

**Предпосылки исследования:**

Тюльпаны—одни из самых известных геофитов, но их таксономия остается запутанной, а их эволюционная история—малоизученной. В этой работе, мы используем изучение пластидного генома для оценки современной таксономии, понимание характера диверсификации рода, и выявление потенциальных видов, являющихся предками культиваров.

**Методы:**

Мы собрали большое количество образцов тюльпанов из живых коллекций, гербариев, онлайн‐источников, и в ходе обширной полевой работы, для того чтобы составить филогению максимального соответствия пластидных геномов рода Tulipa (Liliaceae), представляющих ~86% принятых видов, наряду с видами, иногда принимаемыми в качестве синонимов. Сначала мы использовали программу BEAST и вторичные калибровочные точки для датирования филогенетического дерева, а затем оценили биогеографическую историю рода с помощью программы BioGeoBEARS.

**Основные результаты:**

На основании полученных результатов мы описываем пятый подрод‐ Eduardoregelia и выявляем ряд видов, для которых наши данные не согласуются с современной таксономией рода Tulipa. Наши данные показывают, что род отделился от клады, содержащей Amana и Erythronium, около 32,5 млн. лет назад, при этом наиболее недавний общий предок существовал около 22,8 млн. лет назад в регионе в широком смысле Центральной Азии—в период, характеризовавшийся быстрым поднятием горных систем и связанной с этим аридизации климата в регионе. Диверсификация рода Tulipa преимущественно происходила в Центральной Азии, а быстрая эволюционная радиация имела место в течение последних десяти миллионов лет как результат дальнейшего орогенеза. Несколько миграций за пределы исходного ареала происходили в основном через степи Казахстана и России, а также вдоль гор Кавказа, Ирана и Анатолии в восточное Средиземноморье. Мы также получили данные, свидетельствующие о том, что материнская линия садового тюльпана включала несколько видов, в том числе T. scardica и T. suaveolens.

**Выводы:**

Многие виды тюльпанов имеют ограниченное число характерных признаков, что затрудняет их таксономию, что усугубляется их длительным использованием в садоводстве и широким распространением гибридизации, особенно в садах и огородах. Мы представляем современную филогенетическую систематику этого рода, которая поможет решить многие таксономические проблемы, при этом подчеркивая необходимость дальнейшей работы. Мы подтверждаем важность Центральной Азии в эволюционной истории этого рода и закладываем основу для дальнейшего изучения рода Tulipa и более эффективного сохранения диких видов тюльпанов.

Tulips (*Tulipa* L., Liliaceae) are among the most recognizable geophytes in the world, supporting a billion euro industry (Christenhusz et al., 2013a). Nonetheless, the taxonomy of wild tulips is notoriously difficult (Zonneveld, 2009; Veldkamp and Zonneveld, 2012; Christenhusz et al., 2013a), partly due to a long history in cultivation. Linnaeus described the first tulip species in 1753, but currently over 300 taxa have been named (Govaerts, 2022), with new species being described to the present day (Linnaeus, 1753; Rukšāns and Zubov, 2022; Wilson et al., 2022; Moein et al., 2023; De Groot and Zonneveld, 2022, 2024). However, many different authors have proposed these species, leading to a complex entanglement of species concepts and names, with many previously described species since recognized as either synonyms or naturalized cultivars (Hall, 1940; Zonneveld, 2009; Veldkamp and Zonneveld, 2012; Christenhusz et al., 2013a). Even the type of the genus, *T. ×gesneriana*, is now recognized as a complex hybrid of cultivated origin (Christenhusz et al., 2013a). Historically numerous names have been unstable due to lack of typification, although this has now been largely resolved in a nomenclatural overview of the genus by Christenhusz et al. (2013a) and several other works (Marais, 1984; Van Raamsdonk et al., 1997; Eker et al., 2014).

Currently, there are estimated to be 76–106 accepted species depending on the treatment of certain species complexes (Zonneveld, 2009; Christenhusz et al., 2013a; Eker et al., 2024). Although numerous nomenclatural issues have been addressed (e.g., Christenhusz et al., 2013b; Christenhusz and Wilson, 2022), many recognized taxa remain taxonomically uncertain. Several studies have sought to address existing phylogenetic and taxonomic uncertainty (Hall, 1940; Botschantzeva, 1982; Van Raamsdonk et al., 1997; Zonneveld, 2009; Veldkamp and Zonneveld, 2012; Christenhusz et al., 2013a; Eker et al., 2024), which has led to the recognition of four subgenera (Fay et al., 2001; Zonneveld, 2009; Veldkamp and Zonneveld, 2012). The morphological, cytological, and biogeographical basis for these subgenera is well described (Zonneveld, 2009; Veldkamp and Zonneveld, 2012; Christenhusz et al., 2013a; Eker et al., 2024). However, phylogenetic relationships in the genus remain ambiguous including in taxonomic subgenera and especially in the case of historical sections that have been described primarily from morphological traits or cytological data (Zonneveld, 2009; Veldkamp and Zonneveld, 2012; Christenhusz et al., 2013a; Eker et al., 2024) but that have poor phylogenetic support (Christenhusz et al., 2013a; Eker et al., 2024; Dekhkonov et al., 2025).

Previous phylogenetic analyses of tulips have sampled a range of plastid and nuclear markers, microsatellites or simple sequence repeats (SSRs) (Kiani et al., 2012; Türktaş et al., 2013; Christenhusz et al., 2013a; Pourkhaloee et al., 2018; Hajdari et al., 2021; Eker et al., 2024). Most recently, chloroplast genomic data and transcriptomic data has also confirmed some of the known relationships in the broader Tulipeae and the genus *Tulipa* (Dekhkonov et al., 2025; Zhang et al., 2025). These studies found evidence for most known subgenera, but unlike Eker et al. (2024), they did not support any of the sections in any of the subgenera of *Tulipa*. Although these analyses have advanced our understanding of relationships in the genus, they have also suffered from limited taxonomic sampling, with the most complete molecular phylogenies containing only around 32–40 species, or about 35–44% of tulip diversity (Eker et al., 2024; Dekhkonov et al., 2025; Zhang et al., 2025). New sequencing techniques have enabled more substantial data sets, which are increasingly used to explore the relationships within the broader Liliaceae. For example, a recent whole‐plastome phylogenetic study of the genus *Amana*, a close relative to *Tulipa*, led to more strongly supported phylogenetic relationships (Li et al., 2017). Furthermore, plastome‐level studies at higher taxonomic levels have included a subset of tulip species (Givnish et al., 2016; Liu et al., 2017; Kim and Kim, 2018), and the divergence times of tulips and closely related clades have been explored (Allen, Soltis and Soltis, 2003; Huang et al., 2018; Kim and Kim, 2018). The most‐recent common ancestor (MRCA) of tulips is estimated to have arisen between the end of the Oligocene and the early Miocene, specifically around 20.74 million years ago (Mya), somewhere in Eurasia (Kim and Kim, 2018), and most likely East Asia (Givnish et al., 2016). The MRCA of *Amana*, often inferred as sister to *Tulipa*, also originated somewhere in eastern Eurasia where it remains extant (Kim and Kim, 2018). In contrast, *Erythronium*, also closely related to *Tulipa*, is thought to have originated ~24.4 Mya and to have diverged from its common ancestor with *Tulipa* and *Amana* in East Asia around 57.6 Mya (Kim and Kim, 2018). Current evidence indicates that the MRCA of *Erythronium* evolved in North America, suggesting this clade dispersed to the western hemisphere where it diversified (Kim and Kim, 2018) and likely then spread across the northern hemisphere, with populations in western and central Eurasia lost during the ice ages. Although the evolutionary history of the genus *Tulipa* is still to be fully elucidated, previous work suggests that tulips, similar to *Fritillaria*, *Gagea*, and *Lilium* originated and diversified somewhere in Asia (Kim and Kim, 2018; Peterson et al., 2019), with Central Asia a plausible area of origin.

The mountains of Central Asia are a biodiversity hotspot (Critical Ecosystem Partnership Fund, 2016) where over 5000 species of vascular plants occur, with one quarter endemic to the region (Sennikov and Tojibaev, 2021). Orogenesis has been proposed as an important driver of biological diversification (Antonelli et al., 2018; Rahbek et al., 2019) with a complex geological history contributing to high speciation rates in Central Asia (Muellner‐Riehl, 2019). For example, several plant genera of economic importance have their origin or centres of diversity in Central Asia, including *Allium* (Zohary and Hopf, 2000; Yusupov et al., 2022), *Malus* (Cornille et al., 2014), *Prunus* (Groppi et al., 2021), *Pyrus* (Vinceti et al., 2022), and *Eremurus* (Makhmudjanov et al., 2024). Broadly, the historical climatic changes in the region during the last 30 My have shown a trend toward increased aridification and a cooler climate (Lu et al., 2010; Tang et al., 2011). These shifts occurred with the recession of the Paratethys sea (Ramstein et al., 1997) and significant orogenesis (Manabe and Broccoli, 1990) including the uplift of the Qinghai Tibetan plateau (Miao et al., 2012). Today, the region features a mosaic of geological formations and landscapes including large expanses of open grassland, temperate subhumid grassland, and semidesert steppe, intersected by mountain ranges (Hui et al., 2011; Miao et al., 2012). These conditions favor a geophytic life history, which is associated with seasonal habitats and arid, cool conditions (Howard et al., 2019; Tribble et al., 2021). Consequently, Central Asia hosts a large range of geophytes, including many endemic tulip species (Tojibaev et al., 2018; Sennikov and Tojibaev, 2021).

Globally, wild tulips are found across the Mediterranean and western and Central Asia. Species occur in the southern Iberian peninsula, northern Africa, the Levant, Sicily, Greece including Crete and the Aegean Islands, throughout the southern Balkans and southern Ukraine, up into central Siberia, around the Black Sea coast including Crimea and the Caucasus, Turkey, Iraq, Iran, and east into the Central Asian countries, western China, and Mongolia and south to Afghanistan and the Kashmir (Christenhusz et al., 2013a). However, across this geographic range there are two prominent areas of high species diversity, one in Central Asia and a second across Turkey, Iran, and the Caucasus (Hoog, 1973; Botschantzeva, 1982).

Historically, Central Asia has been hypothesized as the origin of tulips (Hoog, 1973; Botschantzeva, 1982), but this hypothesis has not yet been phylogenetically assessed. And a more complete understanding of the relationships between tulips is needed as a foundation to understand the evolutionary history of diversity in the genus, especially because wild tulips are of interest as a potential genetic resource for breeding (Christenhusz et al., 2013a; Orlikowska et al., 2018). New approaches to exploring the evolutionary history of organisms have emerged, with divergence time dating and biogeographical analyses based on phylogenetic relationships driven by ever‐increasing amounts of data (Cardillo et al., 2017; Noben et al., 2017; Seidl et al., 2020). These methods have the potential to provide deeper insights into species diversification, often linking evolutionary patterns to geological events (Zhang and Fritsch, 2010; Kim and Kim, 2018; Ji et al., 2019). Using these methods and contemporary sequencing methodologies, here we undertook a comprehensive phylogenetic analysis of *Tulipa* to better understand where, when, and how tulips evolved.

## MATERIALS AND METHODS

### Taxon sampling

Leaves were collected from 253 populations during multiple field trips in 2019, 2020, and 2021 and supplemented with material from botanical garden living collections and herbarium specimens. For this project, we undertook three expeditions to Kyrgyzstan in 2019, visiting the Batken, Osh, Jalal‐Abad, Talas, and Chuy regions. In 2020, two expeditions to Kyrgyzstan were undertaken focusing on obtaining species of note, including from the previously unsampled Issyk‐Kul region and to the Talas, Jalal‐Abad, and Chuy regions. An expedition to Tajikistan was also undertaken in 2020 that yielded material from the Gorno‐Badakhshan Autonomous Region and the Khatlon, Districts of Republican Subordination, and Sughd regions. In 2021, material was collected in Kyrgyzstan through expeditions to the Jalal‐Abad, Osh, Chuy, and Talas regions with another expedition to Tajikistan, which included the Gorno‐Badakhshan Autonomous Region, Khatlon, Districts of Republican Subordination, and Sughd regions. During this time, coauthors D. Dekhkonov, K. S. Tojibaev, and N. Beshko gathered leaves for many species from across Uzbekistan, A. Hajdari collected leaves in Kosovo, and M. Firat in Türkiye. Our sampling included a Duc van Tol cultivar, which is one of the oldest known cultivars developed in the Netherlands, to explore how wild species relate to this ancestral cultivar. A voucher specimen for each sequenced individual in our study was stored in relevant herbaria (Appendix 1). When living material was obtained from either living collections or from the wild, leaf material was either frozen using liquid nitrogen or dried using silica gel (Chase and Hills, 1991), directly from the source plant.

### DNA sequencing

To extract DNA from leaf samples, we used a modified CTAB protocol (Doyle and Doyle, 1987). DNA quality was assessed using a NanoDrop 2000/2000c spectrophotometer (Thermo Fisher Scientific, Waltham, MA, USA) and agarose gel electrophoresis. All samples that yielded adequate levels of DNA and were not extensively degraded were frozen and then sent for sequencing at Beijing Genomics Institute (BGI) in Hong Kong. At BGI, either DNB SEQ Normal DNA libraries or Low Input DNA libraries were constructed. These constructed libraries were then sequenced through DNB SEQ PE 100 sequencing. Reads were processed through quality control using SOAPnuke (Chen et al., 2018). At least 1.2 Gb of clean data were obtained for each sample. All returned reads were checked for quality using FastQC (Andrew, 2018). This process led to 245 usable data sets.

### Plastome assembly

We generated plastome genomes through the assembler and SPAdes wrapper Unicycler (Wick et al., 2017), specifically using the script map_bwa_and_assemble.sh (https://github.com/NatJWalker-Hale/sequtils). Initially, we extracted only plastome reads from our sequence data using six reference genomes (five Amana and one Erythronium; Appendix S1) copied and concatenated back‐to‐back (Wang et al., 2018) and the Burrows‐Wheeler Alignment Tool (Li and Durbin, 2009). The extracted reads were then assembled into contigs using Unicycler with conservative mode selected. For most samples, the Unicycler assembly produced three contigs representing the LSC region, SSC region, and an IR region. These assembled contigs were then scaffolded together by mapping them to the first assembled plastome using the function map to reference on Geneious Prime 2020.2.5 to ensure they were all oriented in the same direction because different chloroplast haplotypes can occur in cells (Wang and Lanfear, 2019). In this process, the inverted repeat was duplicated, and the two versions orientated. For 19 of the 245 assemblies, four or more contigs were generated, most likely because of a repeat region that the Unicycler programme could not bridge. In these cases, we mapped the reads to a manually assembled plastome using the function map to reference on Geneious Prime and scaffolded them together, but in two cases, large gaps remained in the resulting assembly. For these two cases, the initial SPAdes‐assembled contigs and the raw reads that had mapped to the plastid were used to generate a consensus sequence, which in turn was used to manually fill these gaps in, where coverage of individual bases was greater than three and the consensus had over 95% similarity to the closest assembled relative.

### Plastome annotation

We annotated a single tulip plastome de novo (*Tulipa binutans* [Code 1 in Appendix 1]) using GeSeq (Tillich et al., 2017). We then aligned this annotated sequence with five reference genomes from GenBank (Appendix S2) that represent five different research studies and five different annotations. Using this alignment, we manually altered incorrectly labelled start and stop regions and named each of the marked de novo genes on Geneious Prime 2020.2.5. This process created a fully annotated tulip plastome where reference genomes were used to accurately position genes, while the de novo annotation was used where discrepancies occurred in the references. This fully annotated plastome was then used as a reference to annotate all other plastomes on Geneious Prime using the application Annotate from with similarity set to 80%. Each subsequent annotated plastome was assessed for inaccurate start and stop codons and for any other errors or structural changes that may have occurred. Several *Tulipa* plastomes were also downloaded from GenBank and their annotations edited to match that of the assembled plastid genomes (Appendix S3). In addition, plastomes for five *Amana* specimens, two *Erythronium* specimens, and two *Gagea* specimens were downloaded from GenBank and annotated using the *Tulipa* reference (Appendix S3). These non‐tulip plastomes were prepared so that they could be used to root the phylogenies inferred using plastome data. Gene maps were produced using OGDRAWv1.3.1 (Greiner et al., 2019). We deposited all our assembled and annotated plastomes in GenBank (BioProject ID: PRJNA1017141, Appendix 1).

### 35S RNA

Genome skimming methods also generate enough reads to produce the genetic loci 5′ETS, 18S, ITS1, 5.8S, ITS2, 26S, and 3′ETS of the nuclear genome sometimes referred to as the 35S rDNA region of the genome (Wu et al., 2020). The 35S region is generally around 5800 bp long in *Tulipa* (Wilson, 2023). The most closely related reference 35S rDNA sequence available on GenBank was *Lilium tsingtauense* (KM117263), so we selected to use this as a reference sequence to assemble tulip 35S rDNA sequences. Initially, we assembled five species 35S rDNA regions, representing the four subgenera with two sequences for the largest subgenus *Tulipa* (Appendix S4). Contigs were assembled from the corresponding raw read data set using SPAdes 3.15.0 before aligning all produced contigs to the *L. tsingtauense* reference in Geneious Prime using the map to reference function. Any mapped contigs were then polished with Pilon v1.23 (Walker et al., 2014) and realigned to the *Lilium* reference again in Geneious Prime using the map to reference function. Contigs with coverage lower than 10 were removed (where the mean per base coverage is 10 or less in a contig), and the remaining aligned contigs were used to generate a consensus sequence using the generate consensus sequence function in Geneious Prime with the strict‐50% threshold selected. We then proceeded to use the five generated tulip sequences as references to extract reads from the raw data set for all other specimens, with the rest of the process remaining the same. All assembled genomes had considerable base heterogeneity outside the 35S rDNA region, so we aligned all sequences with the *L. tsingtauense* reference and transferred the annotations from this reference before removing regions outside the annotations. This process was also used to generate a 35S rDNA sequence for *Amana baohuaensis* using the short read archive available on GenBank (SRR12599520). We deposited all our assembled and annotated 35S rDNA sequences in NCBI GenBank (Appendix 1).

### Phylogenetic analyses

We inferred phylogenies for the plastomes and 35S rDNA using maximum likelihood (ML). The plastome and 35S rDNA regions were initially separated into their corresponding annotated regions and these were extracted in Geneious Prime. The *trnI* and *trnK* intron regions contained unique protein‐coding sequence (CDS) regions of other genes, and so we removed these sections from the *trnI* and *trnK* introns while retaining them in the sequence of the coding genes so that these bases were not duplicated in our analyses. Each extracted region was aligned with the corresponding sequence from all other specimens using MAFFT (Katoh and Standley, 2013) and cleaned using the pxclsq from phyx v1.1 removing columns with 10% occupancy or less (Brown et al., 2017). For both the plastome and 35S rDNA regions, the alignments were then merged into a supermatrix using the pxcat from phyx. In addition, the repetitive regions in the tail end of *trnH–psbA* and *rpl22–rps19* intergenic spacers, which we found to align poorly, were removed. The CDS sequences in the supermatrix were further partitioned based on codon positions. We used iqtree v1.6.12 ModelFinder software (Kalyaanamoorthy et al., 2017) to infer the best partitioning model for each supermatrix using the relaxed hierarchical clustering command with rcluster set to 90 (‐rcluster 90 ‐pre tulipsGTR.merge ‐m MFP+MERGE ‐mset GTR ‐mfreq F ‐mrate G) (Lanfear et al., 2014). However, model selection software was not used to assign an evolutionary model to each partition as it has been deemed unnecessary (Abadi et al., 2019), and so a GTR+F+G model was used for all partitions. The commonly used invariable sites parameter (+I) was excluded because it is known to make it difficult to estimate parameters reliably as its function overlaps with the gamma parameter (+G) (Yang, 2014). Some authors (Gonçalves et al., 2020) have advocated for the use of summary coalescent methods on chloroplast gene trees in light of conflict observed between individual gene trees (Walker et al., 2019), but this practice is criticized on the basis that the plastid is typically inherited as a single linked unit (Doyle, 2022), and therefore, we adopted a concatenation approach here. For each data set, we then inferred a phylogeny using RAxML‐NG v1.01 (Kozlov et al., 2019) with 500 nonparametric bootstrap replicates. The inferred phylogenies were inspected for obvious errors in specimen position, specifically where specimens under accepted species names fell away from other specimens of the same species, which had grouped together or in a clade formed of multiple specimens of another species. We also reviewed the position of previously unidentified specimens that were included in the analysis. A list of clear misidentifications and specimens that remained unidentifiable was compiled, and all trees were pruned of these tips (Appendix S5). In addition, to assess the concordance of nuclear and plastome data, we used the function cophylo in the R package phytools (Revell, 2012) to compare tree structures inferred from these different data. Mismatches between the 35S rDNA and plastome phylogeny were then individually investigated.

### Molecular dated phylogeny

We chose to use full plastome data to infer a starting tree for the molecular dating approach. We decided to remove several specimens from our broader data sets based on their obscure position in the 35S rDNA and plastome inferred trees relative to current taxonomy, often confounded by their collection location being outside of the natural range of the species (Everett, 2013; Christenhusz et al., 2013a) and where there was no way to validify the species name of the specimens from existing material. This included the removal of the *T. maximowiczii* specimen as, although it is likely a true species, the cytonuclear discordance observed in this analysis makes its position uncertain. This process left 80 specimens to be retained in the species phylogeny (Appendix S6). To remove specimens, we initially relabelled sequences in alignments using the pxrls tool from phyx, before removing selected duplicates of species from the alignments using the pxrms tool from the same phyx toolbox (Brown et al., 2017). These alignments were then realigned using MAFFT and cleaned using the tool pxclsq in phyx with columns having less than 10% occupancy removed. A supermatrix of markers was created using pxcat from the phyx package with all CDS markers partitioned further based on codon position. A best scheme partition model was developed for the supermatrix with all partitions assigned the GTR+F+G site model. A species phylogeny was inferred using RAxML‐NG with 500 nonparametric bootstrap trees generated. This phylogeny was then adapted to make branch lengths proportional to time to serve as a starting tree for Bayesian divergence time inference by using penalized likelihood in the function chronos in ape 5.0 (Paradis and Schliep, 2019) implemented in R version 4.2 (R Core Team, 2022), with a correlated clock model and lambda = 0.1. This phylogeny was used as a starting tree for the dating process.

Given the similarity in topology between the CDS and full plastome inferred trees and the computationally intensive nature of Bayesian analyses, we selected to use CDS only data for dating. Due to our limited calibrations (Angelis et al., 2018), we conservatively elected to use a single partition. This data set was filtered to retain only the 80 specimens selected to represent species (Appendix S6), converted to a NEXUS file using the phyx tool pxs2nex, and imported into BEAUti where an XML file was generated that specified the substitution model as a GTR+F+G model, an; uncorrelated relaxed lognormal clock model and a Yule tree prior. Other priors were left as defaults. Additionally, the prepared starting tree was input to fix the topology of the dated phylogeny. Secondary calibration information from Kim and Kim (2018) was used to calibrate Bayesian divergence time inference with node age priors; specifically, we matched our root node and the crown *Tulipa* to their dated tree, which inferred a root (node 1) age of 36 Mya (95% highest posterior density (HPD) = ~23.0–47.0) and a crown age of *Tulipa* (node 2) of 20.74 Mya (HPD = 10.20–23.99). These calibration points were assigned as normally distributed priors with as much of the 95% HPD from Kim and Kim (2018) captured in this distribution as possible. Thus, specifically, node 1 had a sigma of 4.0 and node 2 of 3.5 for their normally distributed priors. MCMC sampling was performed with BEAST v2.6.2 (Bouckaert et al., 2019).

We ran three separate chains for 500 million generations. Parameters and trees were sampled every 1000 generations. The output logfiles of each run were analyzed separately and also combined in Tracer v1.7.1 (Rambaut et al., 2018) to assess convergence of parameters using the estimated sample size (ESS) metric. The ESS of all parameters across all three runs of the BEAST program, and when logs were combined, were all much larger than 200, which is the recommended threshold for suitable modelling. We combined the three separate chains in LogCombiner v2.6.2, with a burn‐in of 25%. A maximum clade credibility consensus tree was generated using a subsample of 100,000 random trees in TreeAnnotator v2.6.2 and visualized in FigTree v1.4.4 (http://tree.bio.ed.ac.uk/software/figtree/). A lineage‐through time analysis was carried out using the function ltt in the R package phytools (Revell, 2012). The rate of species diversification was assessed using a simple gamma statistic built into this function (Pybus and Harvey, 2000) with a Monte Carlo constant rates (MCCR) test approach used to account for incomplete lineage representation using 10,000 simulations with Rho set to 79/96 (the fraction of species represented from all those known) and the function gtt used to assess *γ* across 10 time segments.

### Biogeography

To assess the biogeographical dynamics of *Tulipa*, we divided the global distribution of this genus into a number of subregions. Through this process, we created two maps, one with five subregions, which we refer to as A5 (Figure 4A) and one with six, which we refer to as A6 (Figure 4B). We manually split the natural distribution based on the known distribution directly accounting for the two identified diversity hotspots and the surrounding regions where species occur. Specifically, we ensured for each model that an area covering western Iran, Türkiye, and the Caucasus (in model A5 = D, in model A6 = E) and the key mountain areas of Central Asia were represented (in model A5 = A, in model A6 = B) because these are the two recognized diversity centers for the genus (Botschantzeva,^1982^; Christenhusz et al., 2013a). We then ensured that the Mediterranean (in model A5 = E, in model A6 = F), the northern grasslands (in model A5 = B, in model A6 = C), and Central and South Asia (in model A5 = C, in model A6 = D) were included because these areas are less diverse but have a unique range of tulip species that can be found in similar ecosystem types. The A5 map was therefore designed to split the distribution of *Tulipa* into five relevant regional areas of diversity accounting for: (1) the Mediterranean, (2) Central and South Asia, (3) Turkey‐Iran‐Caucasus hotspot, (4) northern grasslands, and (5) Central Asian hotspot and Far East. The A6 map was similar to the A5 map, except the Central Asian hotspot and Far East was split into two separate regions so as to explore the influence of both these regions in more detail particularly as the far east is much less diverse, but contains many species that are part of the subgenera that diverged first and second from the MRCA of the genus. Maps with more subregions than six were not created due to the computational limitations of the software. We used the R package BioGeoBEARS v1.1.2 (Matzke, 2014, 2018) to carry out the biogeographical analysis with the dated species phylogeny, with the cultivar Duc van Tol tip removed using pxrmt from phyx (Brown et al., 2017) and a text file detailing the species presence across our chosen regions as inputs. According to their natural distribution, we allowed a species to be present in a maximum number of regions of five or six for the A5 and A6 models, respectively, because several known species occupy this broad a range. In addition, we developed a dispersal matrix, which accounted for the limited ability of tulips to disperse pollen or seeds (Kashin et al., 2016), explicitly allowing tulips to only be able to disperse to neighbouring regions. Models were run with and without the constraints of the dispersal matrix. We tested DIVALIKE, DEC, and BAYAREALIKE models as well as these models with an additional founder‐event jump dispersal parameter (+J) (Matzke, 2014). There remains some discussion as to whether the +J parameter is appropriate (Matzke, 2022) or not (Ree and Sanmartín, 2018), so we acknowledge this ambiguity in our results. All models produced were assessed using the Akaike information criterion (AIC).

## RESULTS

### Plastome structure

The 254 tulip plastomes used represent ~86% of the taxonomic diversity in the genus. Annotated plastid genomes were assembled for 245 tulip specimens, plus nine downloaded from GenBank. The assembled plastomes ranged in size from 151,059 to 152,675 bp, and all contained the same 136 genes (Appendix S7). Of these 136 genes, 114 are unique coding regions, plus 22 duplicate genes. These 114 genes consist of 78 CDS sequences, four rRNA sequences, 30 tRNA sequences, and two pseudogenes either with no clear function or a partial sequence (Appendix S8). The GenBank specimens showed a similar complement of genes, with a few gaps noted in some annotations. However, this was likely due to assembly and sequencing issues rather than the outcome of any evolutionary process given the consistency across our much broader plastome data set. There was no clear evidence of gene loss in the tulip plastomes when compared to plastid sequences of closely related *Amana* and *Erythronium* (Li et al., 2017). Nonetheless, we note the *infA* gene region contained several premature stop codons supporting the idea of pseudogenisation of this gene in *Tulipa* (Do et al., 2020).

### Taxonomy

**Figure 1:**
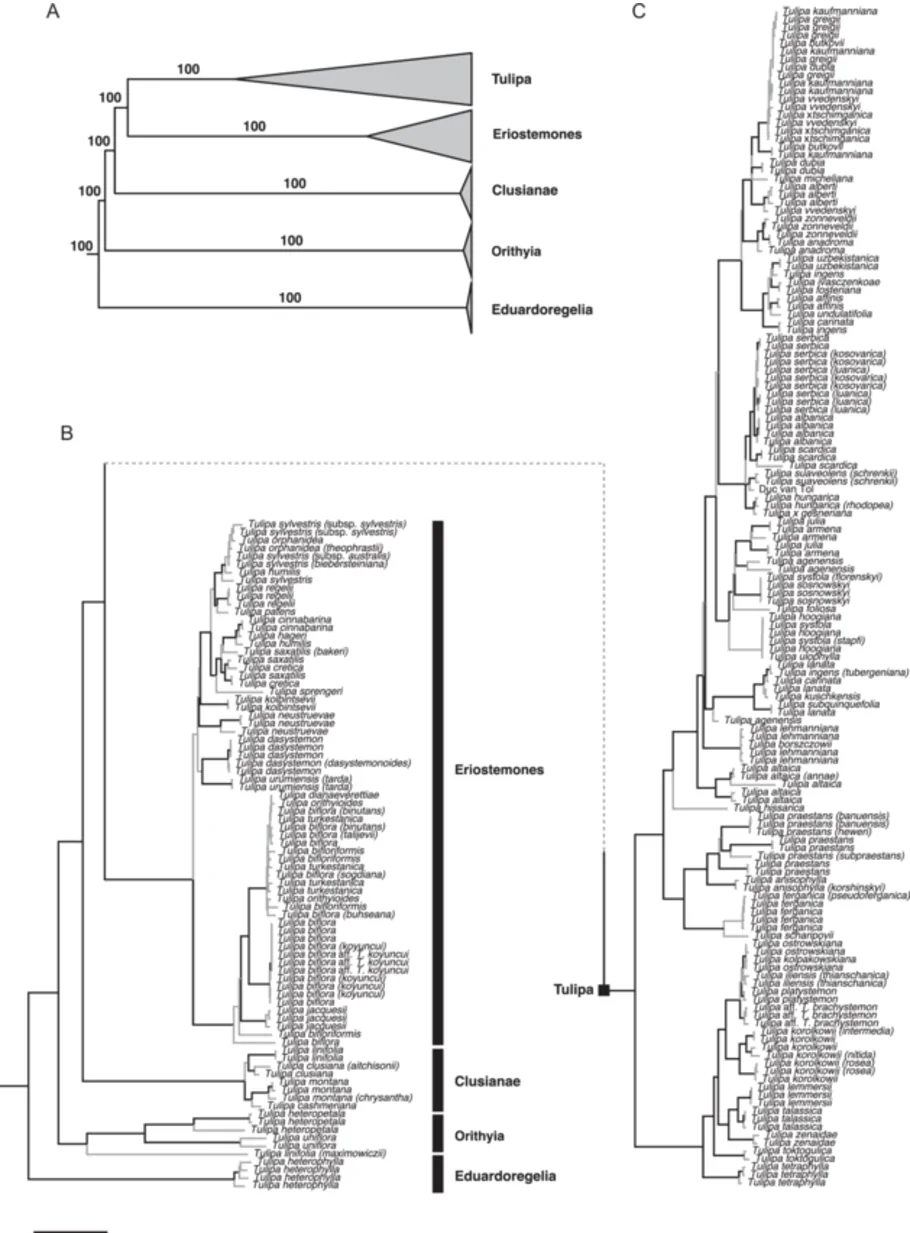
Phylogenetic relationships inferred from plastome data using a maximum likelihood approach. (A) Relationships among the five subgenera as circumscribed and named in this study. Numbers above branches indicate bootstrap support values. (B) Species‐level relationships in the four subgenera Eduardoregelia, Orithyia, Clusianae, and Eriostemones. Black branches represent nodes with 100% bootstrap support; gray branches indicate support values below 100%. (C) Species‐level relationships in the subgenus *Tulipa*. Black branches: 100% bootstrap support; gray: <100% bootstrap support. See Appendix S9 for detailed bootstrap support values.

**Figure 2:**
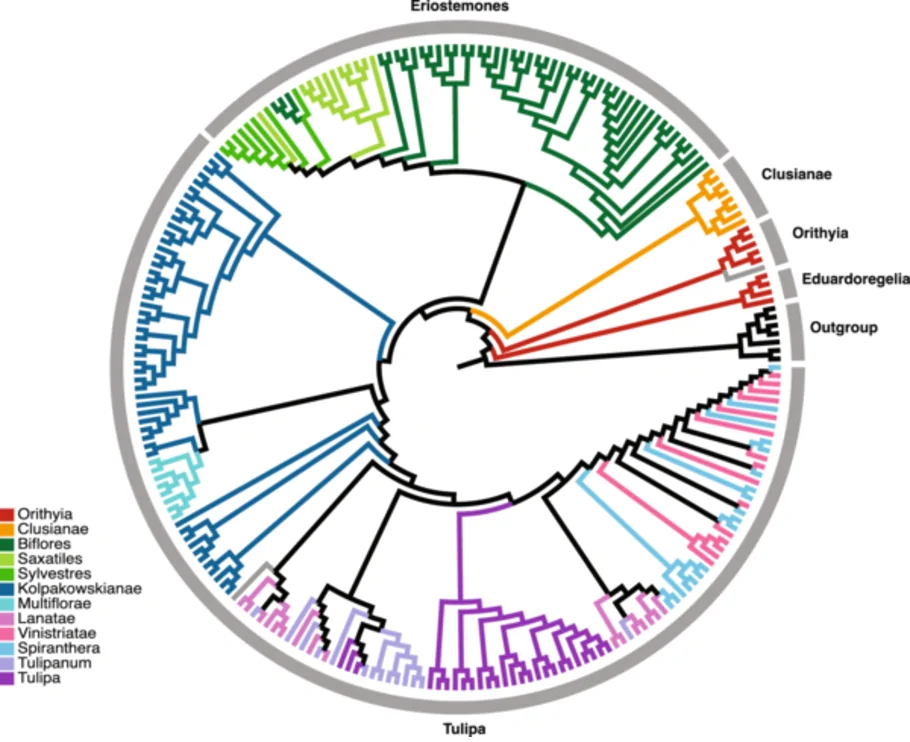
Plastome‐based sectional‐level circular cladogram of the genus *Tulipa*, with outgroup genera *Amana* and *Erythronium*. The five subgenera are indicated by gray circular bars around the perimeter. The 12 sections are color‐coded according to their traditional circumscription, revealing that 10 of the 12 are nonmonophyletic. Grey branches are species with high taxonomic uncertainty.

Our plastome phylogeny confirms the monophyly of *Tulipa* (Christenhusz et al., 2013a) and shows that *Amana* and *Erythronium* are sister (Figure 1; Appendix S9). *Gagea* is supported as the most distantly related genus to *Tulipa* in the tribe Tulipeae, similar to the result of Zhang et al. (2025). The molecular divergence in the genus *Tulipa* and among *Tulipa, Amana*, and *Erythronium* is relatively small compared to the divergence between *Gagea* and other genera of Liliaceae. Phylogenetic relationships in the *Amana‐Erythronium*‐*Tulipa* clade are based on relatively limited sequence variation in these closely related clades. Our phylogenies support the existence of a fifth subgenus (Figures 1, 2) containing the single species *Tulipa heterophylla*, which had been tentatively included in the subgenus *Orithyia* by Christenhusz et al. (2013a). *Tulipa heterophylla* was previously segregated in the genus *Eduardoregelia* Popov, which we here formalize as the name for this new subgenus [*Tulipa* subgenus *Eduardoregelia* (Popov) B.D. Wilson, Shalpykov, Christenh. & Brockington, comb. & stat. nov. Basionym: *Eduardoregelia* Popov, *Index Seminum (AA, Almaatensis)* 3: 9 (1936). Type: *Tulipa heterophylla* (Regel) Baker].

In addition, our plastome‐inferred phylogeny shows that of the currently recognized sections (proposed by Zonneveld,^2009^), only the sections *Clusianae* and *Multiflorae* are monophyletic (Figure 2). In subgenus *Eriostemones*, the three recognized sections *Biflores*, *Sylvestres*, and *Saxatiles* have some topological support, but the placement of several species results in all three sections being nonmonophyletic. In subgenus *Tulipa*, sectional delimitation is extremely convoluted, with only section *Multiflorae* resolvable based on sequence data. However, *Multiflorae* is itself embedded in paraphyletic section *Kolpakowskianae*. A subset of species that make up *Kolpakowskianae* group together into a singular clade, but several species from this section fall outside this group, and so this section is nonmonophyletic, unless it is completely recircumscribed. The remaining five sections are all clearly not monophyletic. These relationships are supported by high bootstrap support at corresponding nodes (>90). This work also indicates that certain taxa previously placed in synonymy (Christenhusz et al., 2013a; Eker et al., 2024) are genetically distinct from the species they were placed under based on their position in the phylogeny and high bootstrap support of corresponding nodes (>90) (Figure 1; Appendix S9). Specifically, this is the case for *Tulipa affinis*, *T. anadroma*, *T. cashmeriana*, *T. hageri*, *T. maximowiczii*, *T. micheliana*, *T. neustruevae*, *T. patens*, and *T. zenaidae*. Here we also tentatively highlight *T. brachystemon*, although there remains uncertainty in the identification of our specimens given their collection location in northern Kyrgyzstan, and we suspect that what we sampled may be *T. jansii* based on the recent description of this species from near that area (De Groot and Zonneveld, 2024). Our work also provides evidence to show the genetic similarity of some species that are currently recognized as separate species and may need to be considered to be merged due to their nonmonophyly, which is supported through high bootstrap support at corresponding nodes (>90). In particular, genetic data shows the close relationships between *T. kosovarica*, *T. luanica*, and *T. serbica*; *T. banuensis*, *T. heweri*, and *T. praestans*; *T. intermedia* and *T. korolkowii*; *T. annae* and *T. altaica*; *T. pseudoferganica* and *T. ferganica*; and *T. biflora* and *T. koyuncui*. We also note that the accession sampled as *T. florenskyi* (as a form of *T. systola*) is genetically similar to *T. sosnowskyi*. In addition, our trees support *T. suaveolens* as the most closely related species to the historical cultivar Duc van Tol (grown in the Netherlands since 1595, one of the oldest cultivars still in cultivation) and that species is likely one of the ancestors of *T. ×gesneriana*, a complex garden hybrid. *Tulipa hungarica* is likely part of this hybrid complex as well given its position in the tree and conservatively should be treated as a “neotulip”, a term used for naturalized forms of *T. ×gesneriana* (see Christenhusz et al., 2013a).

Our phylogenies emphasize the existence of several species complexes, which could not be resolved with our data. These complexes had low bootstrap support for many of the relationships within the complex (<90), but high bootstrap support for the overarching clade (>90) (Figure 1; Appendix S9). One complex is centered around *Tulipa biflora* (subg. *Eriostemones*, clade Biflores), a small, usually white flowered species with a broad distribution and substantial regional morphological variability. Another unresolved species complex centers around a group of Central Asian species in clade Tulipa (subg. *Tulipa*) all of which are primarily large, usually red‐flowered species, including *T. greigii* and *T. kaufmanniana*. Moreover, several highly diverse species were also identified, which were monophyletic and represented by multiple accessions, but with long branches between accessions. These genetically diverse species include *T. altaica*, *T. heterophylla*, *T. korolkowii*, and *T. praestans. Tulipa agenensis* also shows apparently relatively high intraspecific sequence diversity highlighted through the specimens relatively long branch lengths, albeit with only two accessions present in our study. There also remained a range of unidentifiable or obscure specimens that fell in unexpected places with high bootstrap support (>90). The unexpected placement of specimens could not be resolved primarily due to uncertainty in their origin and identification; therefore, these could not be used to make any conclusive taxonomic statements. Of particular note is the isolated position of *T. maximowiczii*, which suggests it may warrant subgeneric status, but better sampling in that clade is required to make such a decision. Further focused analysis is needed to resolve these areas of the phylogenies, but it was beyond the available time and scope of this work.

### Informativeness and discordance between phylogenies

The plastome‐based phylogeny showed higher resolution than the 35S rDNA tree, which broadly had lower bootstrap support values (Appendix S10). Therefore, the plastome inferred tree had more structure to assess subgenera, sections, and species relationships. Importantly, the topology of the 35S rDNA phylogeny and the plastome phylogeny were broadly similar (Appendix S11), suggesting that hybridization and chloroplast capture do not appear to be common processes in this genus, although in the future, nuclear‐based target capture methods may expose more prevalent hybridization especially in species complexes. Our plastome‐based recovery of many commonly regarded species as monophyletic suggest that the plastome can act as an informative marker for tulip taxonomy. Nonetheless, there were several structural conflicts between the topologies. Specifically, the order of the subgenera varied across these two inferred trees (Appendix S11) with the plastome data supporting the existence of a new subgenus *Eduardoregelia*, which is sister to all other subgenera (see discussion on taxonomy), with *Orithyia* diverging off subsequent to *Eduardoregelia*, whereas the 35S rDNA data set supports *Orithyia* as the first diverging subgenus with the new subgenus, as the second diverging branch. Importantly both analyses support the monophyly of *Eduardoregelia*. In addition, the plastome data supports the closer relationship of subgenera *Eriostemones* and *Tulipa* with *Clusianae* sister to this broader clade, while the 35S rDNA phylogeny shows *Clusianae* as more closely related to *Eriostemones*, with subgenus *Tulipa* sister to this pair of subgenera. The plastome‐inferred phylogeny has consistently stronger bootstrap support at these nodes with the 35S rDNA relationships weaker supported, but there still remains evidence of some hard incongruence between these data sets (Appendix S11). This incongruence is further shown in the relationship of several species. In the 35S rDNA inferred phylogeny, some species appear monophyletic, whereas in the plastome inferred phylogeny these same species are paraphyletic. Specifically, these include *Tulipa anadroma*, *T. orphanidea*, *T. sylvestris*, and *T. zonneveldii*, with some conflict between the relationship of *T. scharipovii* and *T. ferganica* also noticeable between trees. Ultimately, we cannot be certain whether a complex biological process may be occurring in these areas of the phylogenies that are not represented in the single coalescent history of the plastome (Doyle, 2022), or whether this incongruence is due to a lack of phylogenetic signal in the more slowly evolving and less character dense rRNA data set, or both.

### Dated molecular phylogeny

**Figure 3:**
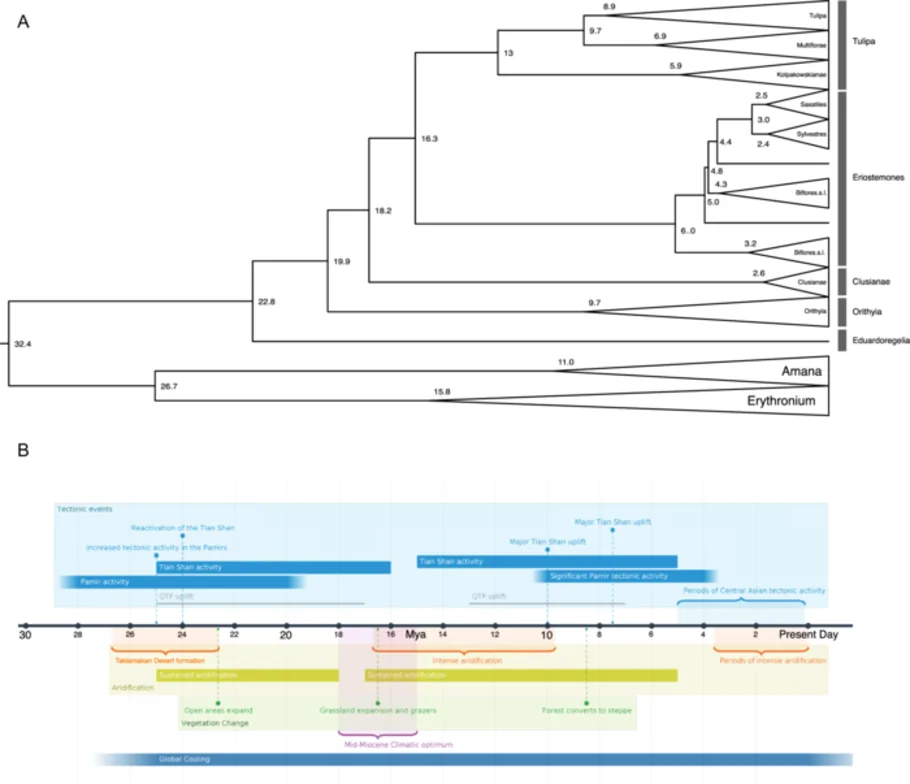
(A) Dated phylogeny of tulips showing subgenera and lower level informal clades (historical sections) with (B) estimated timeline of geologically relevant events summarized from Abdrakhmatov et al., 1996; Atamuradov, 1994; Barbolini et al., 2022; Dercourt et al., 1993; Dupont‐Nivet et al., 2007; Flower and Kennett, 1994; Guo et al., 2002; Hellwig et al., 2018; Hendrix et al., 1994; Hui et al., 2011; Hurka et al., 2019; Kutzbach et al., 1993; Lazarev et al., 2021; Li et al., 2020; Lu et al., 2010; Macaulay et al., 2014, 2016; Manabe and Broccoli, 1990; Miao et al., 2012; Popov et al., 2010; Ramstein et al., 1997; Rögl, 1997; Rustamov, 1994; Sun et al., 2010, 2022; Sun and Zhang, 2008; Tang et al., 2011; Van Baak et al., 2013; Wang et al., 2019; Wesche et al., 2016; Wu et al., 2018; Zachos et al., 2001; Zheng et al., 2015; Zhisheng et al., 2001; Zhongshi et al., 2007.

The MRCA of tulips is inferred to have existed around 22.82 Mya (95% highest posterior density [HPD], 17.99–27.69 Mya), while tulips separated from their nearest relatives around 32.48 Mya (95% HPD, 26.14–39.33 Mya) (Figure 3; Appendix S12). In the plastome phylogeny, newly identified *Eduardoregelia* is the oldest subgenus of *Tulipa* (stem age = 22.82 Mya, [95% HPD, 17.99–27.69 Mya]), with subgenus *Orithyia* the next to diverge (stem age = 19.85 Mya [95% HPD, 15.51–24.36 Mya]), followed by *Clusianae* (stem age = 18.21 Mya [95% HPD, 14.18–22.47 Mya]), until subgenus *Tulipa* diverged from *Eriostemones* (stem age = 16.37 Mya [95% HPD, 12.52–20.22 Mya]). The last two subgenera would later diversify to become the most species rich, while *Eduardoregelia* remained a single species. *Orithyia* diverged into only three species and *Clusianae* into five. For the dated analysis, we adopted the use of informal clades that represent monophyletic groups based on historical sections, although some species have moved between clades based on their phylogenetic position, and we have merged certain sections where there was no topological support for their separation. For the *Eriostemones* subgenus we use the terms *Biflores, Saxatiles*, and *Sylvestres* to represent clades, and for the *Tulipa* subgenus we use clades capturing clearly monophyletic groups of the Kolpakowskianae and Multiflorae sections, while treating all other historical sections in this subgenus as a broad clade called Tulipa. In subgenus *Eriostemones*, clade Biflores diverges first around 6.08 Mya (95% HPD, 4.20–7.92 Mya) with clades Saxatiles and Sylvestres appearing around 3.04 Mya (95% HPD, 2.12–4.03 Mya). The earliest clade to diverge in subgenus *Tulipa* is Kolpakowskianae around 13.11 Mya (95% HPD, 9.89–16.47 Mya), with clades Multiflorae and Tulipa appearing 9.71 Mya (95% HPD, 7.16–12.21 Mya). The dated molecular phylogeny shows that speciation events were infrequent within the first ~13 My of the genus, but in the past ~9 My there has been a rapid increase in speciation across large parts of the tree with the exception of the subgenera *Orithyia* and *Eduardoregelia* (Figure 3; Appendix S12). Explicitly, the lineage‐through time plot and the calculation of the gamma statistic suggest it is appropriate to reject the hypothesis of a constant diversification rate across lineages (*γ* = 2.720253, *P* = 0.0065; Appendix S12). An assessment of the *γ* statistic through time, showed that when the evolutionary timeline of *Tulipa* was split into 10 separate periods (*N* = 10), there was an initial period of diversification, before a period of speciation inertia, with a final period of rapid speciation close to the present time across multiple subgenera (Appendix S12). However, it is difficult to determine whether this recent diversification is due to increased speciation events or decreasing extinction events (Fordyce, 2010). The MCCR test showed that diversification rates were not constant even when accounting for incomplete lineage representation (*γ* = 2.720251 and *P* = 6e‐04). This recent increase in species is likely primarily due to rapid diversification events in subgenera *Eriostemones* and *Tulipa* (Appendix S12). Specifically, the MRCA of subgenus *Tulipa* existed 13.11 Mya (95% HPD, 9.89–16.47 Mya), yet the diversification of this clade mostly took place during the last 9 My with the crown ages of clades Kolpakowskianae (5.89 My [95% HPD, 4.08–7.92 My]), Multiflorae (6.89 My [95% HPD, 4.78–9.23 My]) and Tulipa (8.90 My [95% HPD, 6.65–11.37 My]) the most diverse (Table 1). A similar pattern is mirrored in *Eriostemones*, where the MRCA is estimated to have existed slightly later around 6.08 Mya (95% HPD, 4.20–7.92 Mya) with the MRCAs of clades *Biflores*, *Saxatiles*, and *Sylvestres* diverging 6.08 (95% HPD, 4.20–7.92 Mya), 2.49 (95% HPD, 1.69–3.30 Mya) and 2.43 Mya (95% HPD, 1.47–3.36 Mya), respectively. Subgenus *Clusianae*, although relatively species poor, has also diversified in more recent times after its MRCA existed 2.60 Mya (95% HPD, 1.57–3.75 Mya) (Figure 3). Overall, we observe 92% of the species in our tree diverging from their sister taxa in the last 5 My, emphasizing an extraordinary and recent radiation of this genus across multiple parts of the phylogeny.

**Table 1:** (A) Crown and (B) stem ages of all taxonomically important nodes in the dated phylogeny. HPD, highest posterior density.

Taxonomic group	Mean age (Mya)	95% HPD
(A) Crown ages		
Genus		
*Tulipa*	22.82	17.99–27.69
*Amana*	10.96	7.45–14.76
*Erythronium*	15.88	10.07–22.59
Subgenus		
*Eduardoregelia*	22.82	17.99–27.69
*Orithyia*	9.73	5.86–13.74
*Clusianae*	2.60	1.57–3.75
*Tulipa*	13.11	9.89–16.47
*Eriostemones*	6.08	4.20–7.92
Clade		
Sylvestres	2.43	1.47–3.36
Biflores	6.08	4.20–7.92
Saxatiles	2.49	1.69–3.30
Kolpakowskianae	5.89	4.08–7.92
Multiflorae	6.89	4.78–9.23
Tulipa	8.90	6.65–11.37
(B) Stem ages		
Genus		
*Tulipa*	32.48	26.14–39.33
*Amana*	26.69	19.38–34.22
*Erythronium*	26.69	19.38–34.22
Subgenus		
*Eduardoregelia*	22.82	17.99–27.69
*Orithyia*	19.85	15.51–24.36
*Clusianae*	18.21	14.18–22.47
*Tulipa*	16.378	12.52–20.22
*Eriostemones*	16.37	12.52–20.22
Clade		
Sylvestres	3.04	2.12–4.03
Biflores	6.08	4.20–7.92
Saxatiles	3.04	2.12–4.03
Kolpakowskianae	13.11	9.89–16.47
Multiflorae	9.71	7.16–12.21
Tulipa	9.71	7.16–12.21

### Biogeographic models

**Figure 4:**
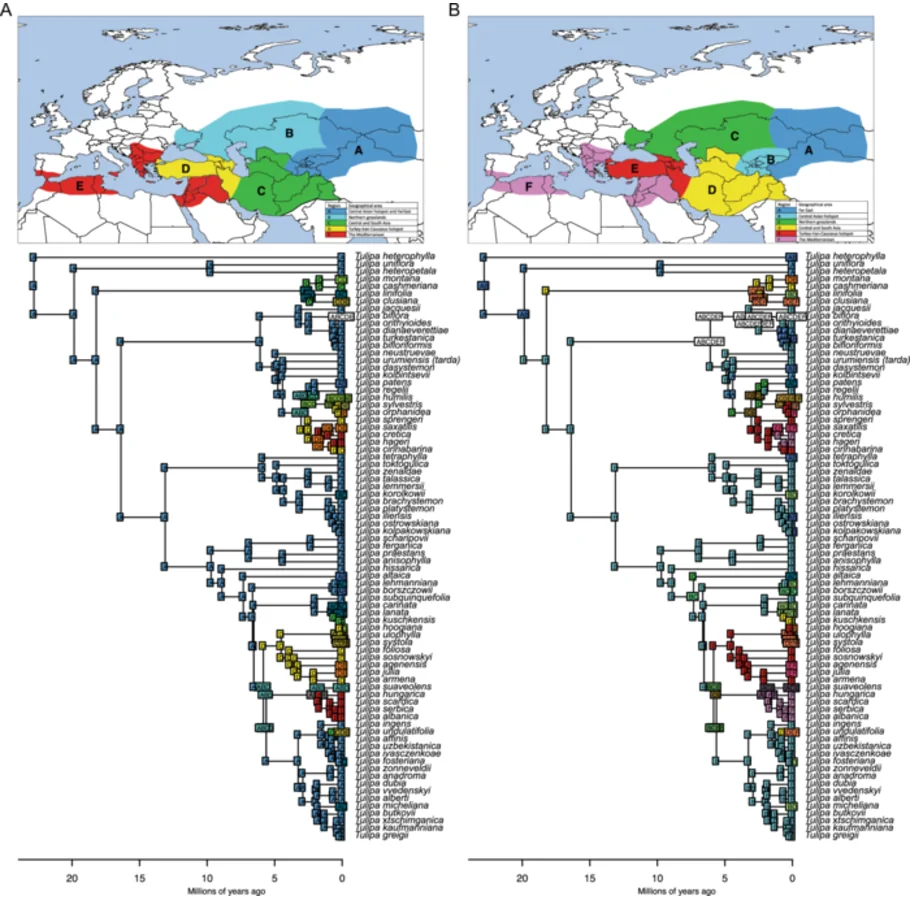
Best‐fit biogeographic models inferred using BioGeoBEARS. (A) Results from the DEC model, with *Tulipa* distribution divided into five areas: Central Asian hotspot and Far East, Central and South Asia, northern grasslands, the Mediterranean, and the Turkey‐Iran‐Caucasus hotspot. (B) Results from the DEC+J model, in which the distribution was divided into six areas: Central Asian hotspot, Central and South Asia, Far East, northern grasslands, the Mediterranean, and the Turkey‐Iran‐Caucasus hotspot. Models were selected based on the lowest AIC scores.

Overall, the DEC or DEC +J models in both the A5 and A6 biogeographical models, had lower AICs (and corrected AICs) than the other models implementable in BioGeoBEARS (Table 2). In addition, the AICs of models inferred with a user‐specified dispersal matrix were the lowest, showing that range expansion into neighboring areas via land connections primarily accounts for the dispersal of tulip diversity. Notably, the DEC model performed better without the founder‐event jump dispersal parameter (+J) in the A5 model implementation, with the +J function converging on zero in the A5 DEC +J model. For the A6 model implementations, the DEC +J model was deemed the most informative, albeit with marginal difference in AIC between DEC and DEC +J models. This marginal difference was likely due to the higher number of parameters rather than anything biologically meaningful. Here, we focus on the results of the A5 DEC model, with some comparison to the A6 DEC +J model with respect to the MRCAs of the subgenera (Figure 4). The biogeographic models show that the MRCA of tulips evolved in the broader Central Asian hotspot and Far East region (Figure 4). The models show that the subgenus *Eduardoregelia*, which is sister to the rest of the genus *Tulipa*, likely evolved only in the Far East region with *Tulipa heterophylla* spreading to the broader Central Asian hotspot and Far East region over time. The MRCA of the *Orithyia* subgenus and the rest of the tulips also likely existed in this broader area, but *Orithyia* tulips became endemic to the Far East and now cannot be found in the Central Asian hotspot, whereas the ancestor of all other tulips became endemic to the Central Asian hotspot at this time. The branching of the *Clusianae* tulips from this ancestor appears to have led to their MRCA evolving in the Central and South Asia region from which several species, such as *T. montana* and *T. clusiana* expanded their range farther west into the Turkey‐Iran‐Caucasus hotspot and *T. clusiana* then onto the Mediterranean potentially aided by humans, while *T. linifolia* appears to have reoccupied parts of the Central Asian hotspot albeit currently only known from parts of southern and central Tajikistan (Figure 4).

**Table 2:** The Akaike information criterion for models run in BioGeoBEARS with a dispersal matrix.

	Five areas (A5)		Six areas (A6)	
Model	AIC	AICc	AIC	AICc
DEC	316.3	316.4	406.4	406.6
DEC +J	318.1	318.4	406.2	406.5
BAYAREALIKE	375.5	375.6	450.9	451.1
BAYAREALIKE +J	341.1	341.5	428.2	428.5
DIVALIKE	345.5	345.7	442.6	442.8
DIVALIKE +J	347.1	347.4	438.8	439.2

The MRCA of the *Eriostemones* and *Tulipa* subgenera occupied the Central Asian hotspot where it diverged to create these two subgenera both of which had MRCAs that occurred in this region as well. Yet, the MRCA of the *Eriostemones* subgenera spread rapidly leading to the Biflores clade that centers around the widespread species of *T. biflora*, which now occurs in all regions of the distribution of the genus, but many taxa in the Biflores clade remain endemic to the Central Asian hotspot and Far East. This widespread MRCA also led to a later ancestor evolving in the northern grasslands leading to a clade containing the species of the Sylvestres and Saxatiles clades that now occupy the Turkey‐Iran‐Caucasus hotspot and the Mediterranean such as *T. saxatilis*, *T. sylvestris*, and *T. cretica* (Figure 4). Subgenus *Tulipa* primarily diversified in the Central Asian hotspot with species of the Kolpakowskianae clade remaining nearly all endemic to this area, although the range of *T. korolkowii* now includes Central and South Asia and *T. iliensis* and *T. kolpakowskiana* can be found in the Far East also. The next branch of *Tulipa*, the Multiflorae clade, also diversified but remained endemic to the Central Asian hotspot. After that, several groups in the broader Tulipa clade emerged that occupy several distinct regions of the *Tulipa* distribution. One group diverged, containing species such as *T. borszczowii*, *T. altaica*, and *T. carinata*, which centers around the Central Asian hotspot, but many of the geographical ranges of these species reached into either the northern grasslands or into Central and South Asia. Another group diverged here with an MRCA that spans the Central Asian hotspot and the northern grasslands, which then branched into two clades. One of these has an MRCA in the Turkey‐Iran‐Caucasus hotspot where it diversified into many of the species known from this region such as *T. foliosa* and *T. systola*, with some species such as *T. julia* reaching the Mediterranean region. The remaining group then diverged into two with one containing species that went on to reach the Mediterranean and diversify into many of the Balkans species, and the final one had an MRCA endemic to the Central Asian hotspot where it diversified rapidly into many of the Central Asian species known from the Tulipa clade (Figure 4).

## DISCUSSION

### Taxonomic insights

Our work confirms the genus *Tulipa* as a monophyletic group, with *Gagea* as the most distantly related genus in tribe Tulipeae, and a clade containing *Amana* and *Erythronium* as sister to *Tulipa*. The clades that form *Amana*, *Erythronium*, and *Tulipa* are separated by relatively limited genetic variation and could be treated as a single genus were it not for the wide usage of these genus names in horticulture (Christenhusz et al., 2013a). The genus *Tulipa* has previously been subdivided into four subgenera based on molecular data (Fay et al., 2001; Veldkamp and Zonneveld, 2012; Christenhusz et al., 2013a; Eker et al., 2024) and genome sizes (Zonneveld, 2009; Veldkamp and Zonneveld, 2012). Both our plastome and 35S rDNA validate these four subgenera, but also indicate that a fifth subgenus should be recognized. This subgenus is named *Tulipa* subgenus *Eduardoregelia* and contains only a single species, *Tulipa heterophylla*. This species was originally described as *Orithyia heterophylla* Regel, due to the presence of a style on top of its ovary. It produces only two leaves and has a yellow flower, all characteristic of subgenus *Orithyia* (Fay and Christenhusz, 2013). However, this species is morphologically distinct from the other species of *Orithyia* because of its drooping flower reminiscent of *Erythronium*, which is a good diagnostic morphological trait of this new subgenus.

Before our analysis, the genus had 12 accepted sections (Zonneveld, 2009), with each subgenus consisting of one or more sections. Subgenus *Eriostemones* was separated into sections *Biflores*, *Saxatiles*, and *Sylvestres*, and subgenus *Tulipa* was divided into sections *Kolpakowskianae*, *Lanatae*, *Multiflorae*, *Spiranthera*, *Tulipa*, *Tulipanum*, and *Vinistriatae,* while subgenera *Clusianae* and *Orithyia* contained only single sections. Our study does not find any of the sections in the subgenera *Eriostemones* and *Tulipa* to be monophyletic except one, *Multiflorae*. For instance, there is evidence that *Tulipa regelii* is part of *Sylvestres* rather than *Biflores*, which aligns with previous morphological and phylogenetic analysis of this species (Christenhusz et al., 2013a, Eker et al., 2024). The position of *T. hageri* in our tree would lead to it being recognized as part of *Saxatiles*, but this positioning breaks the link to its morphologically similar species *T. orphanidea* under which it was previously placed, which would remain part of section *Sylvestres*. *Tulipa humilis* is paraphyletic in our phylogeny with specimens falling in both *Sylvestres* and *Saxatiles*, suggesting that the morphological circumscription of these sections is challenging and that there is considerable uncertainty around the identity of these specimens; hence, we have not drawn any conclusions from their phylogenetic positions. In addition, we observe that section *Biflores* is paraphyletic with *T. dasystemon*, *T. kolbintsevii*, *T. neustruevae* and *T. tarda* appearing on a clade sister to a broader clade that contains *Saxatiles* and *Sylvestres* contradicting previous studies (Eker et al., 2024). Their position here is weakly supported by bootstrap values, and therefore to re‐circumscribe or to accept any sections in *Eriostemones*, we need additional data. Nonetheless, due to the presence of nonmonophyletic sections in the subgenus *Eriostemones*, we do not think that it warrants formal subdivision at this point. The sections of subgenus *Tulipa* are even more convoluted than in subgenus *Eriostemones*. Even though they were distinguishable based on genome size (Zonneveld, 2009), they do not hold when using molecular phylogenetic studies, indicating that genome size is not necessarily an indication of relationship. Only section *Multiflorae* can be resolved based on sequence data, but this section is embedded in the paraphyletic *Kolpakowskianae*. *Tulipa altaica*, *T. anisophylla*, *T. annae*, *T. borszczowii*, *T. ferganica*, *T. hissarica*, *T. lehmanniana*, and *T. scharipovii* all fall outside this cluster. The remaining species fall in a broader clade that encompasses the remaining five sections of Zonneveld (2009). None of these sections are monophyletic, and all members of former sections *Lanatae*, *Spiranthera*, *Tulipanum*, and *Vinistriatae* are intermingled with members of section *Tulipa*. Given the general lack of sectional monophyly, we have abandoned the usage of sections in the genus *Tulipa* altogether and recognize only subgenera, which aligns broadly with the results of a number of previous morphological and phylogenetic studies (Christenhusz et al., 2013a; Eker et al., 2014, 2024; Dekhkonov et al., 2025; Zhang et al., 2025). In theory, it would be possible to maintain some sections, but these would need to be completely re‐circumscribed, losing the historical link to the original circumscription. Re‐circumscribing sections is likely to cause more confusion than clarity for future users of these names, and it is therefore better to use informal clade names below the level of subgenus.

As part of this work, we have identified a number of taxa where our phylogenetic tree contradicts existing taxonomy (Zonneveld, 2009; Christenhusz et al., 2013a; Dekhkonov et al., 2023; Eker et al., 2024; Kubentayev et al., 2024). There has continued to be expansion of the genus in recent years due to an influx of new species described, including a new species from an earlier output of our work (Wilson et al., 2022). Of the species newly described since 2013 and included in our study, we can shed light on some of their relationships. *Tulipa jacquesii* (Zonneveld, 2015) is well supported as a clade sister to the *T. biflora* complex when considering only highly supported bootstrap branches. The *T. biflora* complex itself is polyphyletic with *T. koyuncui* (Eker and Babaç, 2010) and *T. dianaeverettiae* (De Groot and Zonneveld, 2020) deeply embedded in it. We highlight that these species are clearly not phylogenetically separate from *T. biflora* in our work; they show limited morphological variation from *T. biflora*, their ranges overlap with the broader *T. biflora*, and there are known intermediates of *T. biflora* and *T. koyuncui* some of which we sampled in our research under the name *T. biflora* aff. *koyuncui*. Apart from color, *T. koyuncui* differs in having only slightly shorter inner and outer filaments, which overlap with the lower end of the *T. biflora* measurement range for this trait. While *T. dianaeverettiae* also sits in the geographical range of broader *T. biflora* (albeit at the edge) and is morphologically described based on slightly fewer hairs on the leaf edges and stem than *T. biflora*, this slight trait difference may be natural variation given the limited sampling of *T. biflora* from across its range. *Tulipa biflora* remains polyphyletic with several accessions of *T. biflora* appearing in a clade with *T. bifloriformis*, *T. orithyioides*, and *T. turkestanica*. The new species *T. kujukense*, *T. lorestanica*, and *T. salsola* (Rukšāns and Zubov, 2022) also likely belong in this group, but no material of the last three species was available for study. There remains considerable uncertainty surrounding many of these species, including these newly described taxa, because they fall in the broader range of *T. biflora*, clearly share most if not all morphological traits with this species, and the natural variation in many of the diagnostic traits used to describe these taxa are poorly understood in the broader *T. biflora*. It is clear that further work, preferably using population dynamic techniques, needs to be undertaken on this complex across its range in the wild to resolve the many remaining taxonomic issues, and we believe retaining a broad concept of *T. biflora* will be the best course of action until this can be conducted.

In our work, the recently described *T. ivasczenkoae* (Epiketov and Belyalov, 2013) is found to be sister to *T. fosteriana*, *T. toktogulica* (Wilson et al., 2022) is sister to a broader clade containing species such as *T. korolkowii* and *T. talassica*, and *T. zonneveldii* (De Groot and Tojibaev, 2017) is sister to *T. anadroma*. Our results support previous morphological descriptions (e.g., Everett, 2013) that accept these as valid species. *Tulipa intermedia* (Tojibaev et al., 2014) originally considered to be morphologically intermediate between *T. scharipovii* and *T. talassica* unexpectedly resolved in the clade of *T. korolkowii*. On review of its range and habitat type, this taxon appears to exist within the potential range of *T. korolkowii* and *T. scharipovii* (Wilson et al., 2021; Tojibaev et al., 2022), but not that of *T. talassica*. Morphologically, it shares many traits with *T. korolkowii* especially *T. intermedia* var. *korolkowioides*, which clearly has an almost identical flower. We highlight that further work is needed to identify if it is truly different from *T. korolkowii* and here suggest abandoning the use of varieties given they are mainly based on flower color, a trait known to be notoriously variable within tulip species (Tojibaev et al., 2014). *Tulipa annae* (De Groot and Zonneveld, 2020) fell in the clade of *T. altaica*, a species with which it clearly overlaps in range (Kubentayev et al., 2024) and shares many morphological traits albeit several differences are noted in morphology—primarily the presence of hairs on the inside and outside of the lower base of the tepals and on the surface of the leaves (Everett, 2013; De Groot and Zonneveld, 2020). *Tulipa pseudoferganica* resolved in the clade of *T. ferganica*, which was somewhat unsurprising given its clear morphological similarity and range overlap. There was uncertainty around the species identity of the material we tentatively labeled as *T. brachystemon*, and we note that it may have been a new subpopulation of *T. jansii*, a species that was only recently described (De Groot and Zonneveld, 2024). Regardless of this uncertainty, the sampled material reflected a clearly monophyletic taxon, and so we highlight here that more work is needed to determine the range and taxonomic validity of both of these species especially given recent work highlighting the similarity of *T. brachystemon* and *T. kolpakowskiana* (Almerekova et al., 2024).

In addition, from our phylogenies, we have considerable evidence that several previously synonymized species may in fact warrant recognition as species based on their separation from the taxa under which they were synonymized based on morphological grounds. Specifically, we found evidence to support the reinstatement of *Tulipa affinis*, *T. anadroma*, *T. cashmeriana*, *T. maximowiczii*, *T. micheliana*, *T. neustruevae*, *T. patens*, and *T. zenaidae*. We note here that in several of these cases, these taxonomic insights are in line with morphological analyses by Eker et al. (2024) in which *T. brachystemon*, *T. neustruevae*, *T. patens*, and *T. zenaidae* were accepted. In addition, our phylogenetic evidence shows that what is being considered as *T. micheliana* across much of Central Asia is not closely related to *T. undulatifolia* under which it was previously considered a variety (Christenhusz et al., 2013a; Eker et al., 2024). We thus highlight that a range of cryptic species may occupy different biogeographical distributions under the name *T. undulatifolia*, especially given the extremely broad range of this species as currently recognized. We therefore found evidence that could support the reinstatement of *T. micheliana*, which aligns with the common use of the name *T. micheliana* by Central Asian researchers for a clearly identifiable species, but we highlight that further exploration of *T. undulatifolia* and its closely related species is needed. It should be noted that in horticulture the names *T. micheliana*, *T. eichleri* and *T. undulatifolia* are frequently confused and that it includes also hybrids with other species. *Tulipa hageri* was positioned outside *T. orphanidea* under which it is currently synonymized, falling sister to *T. cinnabarina*. We emphasize that more information is needed to confirm or refute its species status alongside a broader review of the *T. orphanidea* complex, which is polyphyletic in our tree.

We also found evidence that strongly supports the synonymization of several taxa. For instance, in our tree *T. kosovarica* and *T. luanica* are found in a polyphyletic complex with *T. serbica*, which suggests that these are all one species as highlighted in previous phylogenetic and morphological work (Hajdari et al., 2021; Eker et al., 2024). We also found evidence that *Tulipa banuensis* and *T. heweri* are phylogenetically indistinguishable based on plastid data from *T. praestans* with which they have also been historically considered morphologically similar (Everett, 2013). Specifically, *T. heweri* has been considered an albino form of *T. praestans* and *T. banuensis* as a form of *T. praestans* without the blotch in the flower. They therefore could warrant synonymization, although we recognize the need for further work to unravel the *T. praestans* complex given the morphological and phylogenetic diversity captured within it. We also found evidence that suggests that *T. hungarica* could be treated as a neotulip under *T. ×gesneriana*, given their close relationship in our phylogeny, but suggest further exploration is needed here. Furthermore, we highlight that *T. annae*, *T. koyuncui*, and *T. pseudoferganica* all fell in the broader clade of *T. altaica*, *T. biflora*, and *T. ferganica*, respectively, highlighting the phylogenetically close relationships of these taxa based on the plastid marker, which could support their synonymization. We also note that *T. florenskyi* could be a synonym of *T. sosnowskyi* given its position as sister to this species, rather than *T. systola* where it was tentatively placed based on morphology by Christenhusz et al. (2013a), and that *T. borszczowii* appears to potentially warrant being placed under *T. lehmanniana*.

The identification of wild tulip species is often difficult due to the broad variation of morphology even within species, leaving few taxonomically informative traits (Zonneveld, 2009). From our experience, we know that tulip species in living collections, herbaria, and online databases are frequently misidentified and that, even in the field, comprehensive taxonomic expertise is needed to identify specimens. Errors in identification are easily made, especially when details of the bulb are absent. It is clear that some taxa are more commonly mistaken than others, and special care is needed in particular areas of identification. Given the taxonomic complexity of this genus, we expect that the total number of accepted species is likely to change again in the near future as new species continue to be described based on morphology and accepted species and synonyms are reassessed, particularly in light of new molecular techniques that are being developed. Already our phylogeny highlights several areas where further analyses are clearly needed that could lead to taxonomic changes. These primarily correspond to species complexes, genetically diverse species, potential hybrids and several specimens treated as synonyms of accepted species, but that did not fall in expected clades. We highlight that, in spite of existing taxonomy, a number of species (e.g., *T. armena*, *T. humilis*, *T. julia*, *T. lanata*, *T. orphanidea*, *T. saxatilis*, *T. sylvestris*) remain nonmonophyletic in our phylogeny. These are likely part of species complexes that may need a population genetic approach (e.g., Hyb‐Seq or RADseq; Davey and Blaxter, 2010; Dodsworth et al., 2019) to tease apart these taxa, but this work was beyond the scope of the current study. In particular, we note other species and areas that need further investigation: several polyphyletic species that group around *T. biflora*, *T. lanata*, *T. greigii*, *T. kolpakowskiana*, and *T. systola* on short branches; the considerable taxonomic uncertainty around species such as *T. undulatifolia*, where we have made hesitant interpretations based on the phylogenetic position of a single specimen that is not necessarily reliable; and areas of cytonuclear discordance including that of *T. maximowiczii* (sometimes considered a synonym of *T. linifolia*), which our 35S rDNA and plastome data suggests may warrant a separate subgenus, but here we have only highlighted that it is likely a valid species due to our limited sampling of subgenus *Clusianae*.

### The ancestors of tulip cultivars

Our phylogeny highlights several results that may contribute to broader understanding of the tulips used in the initial horticultural breeding of cultivars. Our plastome‐inferred phylogeny shows that one of the oldest cultivars known, Duc van Tol, is closely related to *Tulipa suaveolens* supporting the theory that this species may have been used in the initial breeding of cultivated tulips (Grime and Mowforth, 1982; Kritskaya et al., 2020). Our phylogeny also highlights the close relationship of tulips sampled from the Balkan region with both *T*. ×*gesneriana*, which is known to be an old cultivar of hybrid origin (Christenhusz et al., 2013a), and the Duc Van Tol cultivar suggesting they also likely played a role in the initial cultivation of wild tulips. Importantly, in 1601, Clusius mentioned two types of tulips being sold in Istanbul under the names cafe lale and cavala lale (Clusius, 1601). The name cafe likely links to an early‐flowering tulip from Kefe (now Feodosya) in Crimea, Ukraine, which may be *T. suaveolens*, and cavala refers to a late‐flowering tulip from Kavala a port town in Greece (Mordak, 1990), from where *T. scardica* may have been shipped to Istanbul. Our current data supports the theory that *T. suaveolens* and *T. scardica* may have had a significant role in initial cultivation efforts and highlights the importance of southeastern Europe for early breeding material for tulip cultivars in Turkey. In addition, we note that a specimen of *T*. ×*gesneriana*, is closely related to *T. hungarica*, which could suggest that *T. hungarica* played a role in the history of horticultural tulips, but it more likely is an escaped early cultivar. It is morphologically similar to other neotulips differing merely in flower color. It probably naturalized in this region, likely from Ottoman settlements in the area (Kontler, 1999). Another specimen of *T*. ×*gesneriana—*recognized in this work as a potential misidentification due to its collection in Iran where this taxon is not known to occur*—*is closely related to specimens of *T. agenensis*, a species known to be widely naturalized from cultivation in Europe and Anatolia. Although this specimen of *T. ×gesneriana* is likely not a true representative of this hybrid cultivar, we cannot rule out *T. agenensis* as another potential contributor to cultivar diversity. There is still much ambiguity around which species contributed to the original cultivars, especially with regard to the role of Central Asian species (Christenhusz et al., 2013a), which in more recent times have been used to create new cultivars. However, research is now beginning to highlight a few candidate species as the most important contributors to the first cultivars, with our work adding another layer of information to resolve this question.

### Origin and evolution of tulips in Central Asia

Our dated phylogeny shows that the ancestor of the genus *Tulipa* diverged from a clade that later diversified into *Amana* and *Erythronium* about 32.48 Mya. Previous dated trees show *Erythronium* as sister to *Tulipa* (Christenhusz et al., 2013a), suggesting that this broader split likely occurred in eastern Asia (Kim and Kim, 2018). Our phylogeny shows that *Amana* and *Erythronium* form a monophyletic group, and therefore, this clade is sister to *Tulipa*, with implications for the stem age of *Tulipa*. The MRCA of the genus *Tulipa* had previously been estimated to have originated around 20.74 Mya (Kim and Kim, 2018), but here we support a slightly older origin of 22.82 Mya (Table 1), albeit with statistical uncertainty surrounding both dates. This initial split giving rise to *Tulipa* likely occurred around the Eocene–Oligocene transition (Figure 3; Appendix S13), and our biogeographic models show that the MRCA of *Tulipa* existed in the broader Central Asia region, specifically the Central Asian hotspot and Far East region (Figure 4). We emphasize that the origin of this genus cannot be linked to the present territory of a single country with our A6 model, in which area A of the A5 model was split into two parts, not resolving further the ancestral area of the MRCA than the A5 model. Nonetheless, the broader area of Central Asia was crucial to initial speciation events in this genus, with all the MRCAs of subgenera evolving in the Central Asian hotspot and Far East region between 23 and 16 Mya (Figure 4, Table 1). Our dated phylogeny shows that subgenus *Eduardoregelia* forms the first clade to diverge from the MRCA, with *Orithyia* second around 3 My later, with our biogeographic models projecting that their MRCAs inhabited the eastern zone of Central Asia. The MRCAs of all other subgenera are projected to have originated in the hotspot of Central Asia, with the *Clusianae* clade emerging 1.5 My after *Orithyia* diverged, and finally *Eriostemones* and *Tulipa* separated around 16.38 Mya. Given these timings, and the evident significance of Central Asia in the context of the MRCA of *Tulipa* and more recent species radiations, we next discuss some of the climatic and geological evidence to support these phylogenetic inferences.

We infer that the MRCA of *Tulipa* existed in a period of significant aridification in the broader Central Asia region (Zachos et al., 2001; Tang et al., 2011), when large areas of dry habitat are thought to have already existed (Guo et al., 2002; Sun et al., 2010) and the desertification of the Taklamakan Desert was initiated (Zheng et al., 2015). This aridification coincides with a period of global cooling and the retreat of the Paratethys Sea, which may have contributed to the drying process (Ramstein et al., 1997; Dupont‐Nivet et al., 2007; Lu et al., 2010; Miao et al., 2012; Sun et al., 2022). These climatic conditions have been commonly associated with the evolution of geophyte lineages (Howard et al., 2019). There was potentially also a shift to more open seasonal environments in this transition period with steppe‐like ecosystems already fairly well established (Barbolini et al., 2022), in which tulips are now commonly found. In this period, significant tectonic activity, including mountain formation in the Tien Shan (Hendrix et al., 1994; Macaulay et al., 2014, 2016), some activity in the Pamir region (Wang et al., 2019), and uplift of the Qinghai‐Tibetan plateau (Kutzbach et al., 1993) is thought to have been occurring. This orogenic activity caused changes in climate and rainfall patterns in the region, which again may have further promoted aridification (Manabe and Broccoli, 1990; Hellwig et al., 2018). In addition, this tectonic activity created a mosaic landscape with widely spaced mountain ranges and large intermontane valleys (Macaulay et al., 2014), whilst large areas of the region were likely still covered by the Paratethys Sea limiting habitable areas (Li et al., 2020). Therefore, it is likely that a combination of environmental factors both specific to Central Asia and globally may have played a role in driving early speciation in the genus *Tulipa*. Specifically, we highlight the link between climate and mountain formation in tulip diversification, which aligns well with evidence of increased species richness in mountain areas, where there is interplay between surface uplift, climate change, and atmospheric circulation (Antonelli et al., 2018), with mountain building often linked to a “species‐pump” effect (Muellner‐Riehl, 2019).

The interplay of aridification and orogenesis is also likely associated with the origin of subgenera and more recent species radiations in Central Asia. Diversification of the genus appears to have slowed approximately 16 Mya, around the time of the Mid‐Miocene Climatic optimum. During this period, the step‐by‐step regression of the Paratethys Sea ceased (Dercourt et al., 1993; Rögl, 1997; Zhongshi et al., 2007), and a period of global warming occurred (Flower and Kennett, 1994), which led to changes in ecosystems. Pollen records from Central Asia show an increase in more thermophilous species (Sun and Zhang, 2008) and the expansion of grassland areas (Barbolini et al., 2022). In this period, tectonic activity broadly slowed, and a warmer climate appears to have coincided with increased precipitation in areas of Central Asia until around 13 Mya (Miao et al., 2012). Precipitation levels remained fairly stable in the region until the late Miocene (Miao et al., 2012) before further aridification that was associated with renewed orographic activity (Lu et al., 2010; Tang et al., 2011). Diversification in the genus *Tulipa* seems to have rapidly increased around 9 Mya, especially in the subgenus *Tulipa* in Central Asia, where uplift in the region and subsequent changes in climate likely drove speciation events. Continued aridification (Zhisheng et al., 2001; Lu et al., 2010) and the replacement of large areas of forest with grassland such as steppe (Hui et al., 2011) may have prompted the diversification of *Tulipa*. In addition, across the region, expanses of grassland are thought to have been maintained by large grazing mammals later in this period (Atamuradov, 1994; Wu et al., 2018). Tulips often grow in open areas and geophyte diversity is generally higher under light grazing, although species‐specific effects can vary (Noy‐Meir and Oron, 2001).

In parallel, increased orogenesis, including the emergence of a number of mountain ranges, led to decreasing intermontane basin sizes and further breakup of habitat areas (Macaulay et al., 2014); most of the Tien Shan is thought to have emerged during the last 10 My (Abdrakhmatov et al., 1996). This changing landscape—the increase in grassland and steppe land in Central Asia linked to a broader global expansion of grasses (Barbolini et al., 2022)—and the occurrence of multiple significant drying events, especially during the last 6 My (Sun and Zhang, 2008; Lu et al., 2010), means that Central Asia continued to aridify, while the orographic landscape became more complex. Wild tulips significantly diversified during this period with particularly large radiations in the subgenera *Eriostemones* and *Tulipa* and a relatively smaller diversification in subgenus *Clusianae*. At this time, many new clades and species appeared in Central Asia, while tulip diversity was expanding across the broader Eurasian range of the genus. It is important to note here that our results align well with previous suggestions that Central Asia has several separate centers of speciation contributing to the diversity of the region, specifically the arid deserts of Central Asia, the Tien Shan mountains, and the Pamir‐Alay mountains (Botschantzeva, 1982). Nonetheless, we lack the resolution to assess these specific centers in detail but do highlight that our phylogeny shows that the relationships between species of these centers is not monophyletic, and therefore, they may not be as separate as previously believed. In addition, our study provides some support to the view that the ancestors of tulips were mountain dwelling species, with the older subgenera of *Eduardoregelia* and *Orithyia* containing mostly yellow‐flowered species with star‐shaped flowers that grow in the mountains, but we cannot confirm that the ancestors of tulips occurred in the lower to middle mountain belts and definitely not in high mountain areas as previously thought (Botschantzeva, 1982).

### Dispersal of tulips out of Central Asia

**Figure 5:**
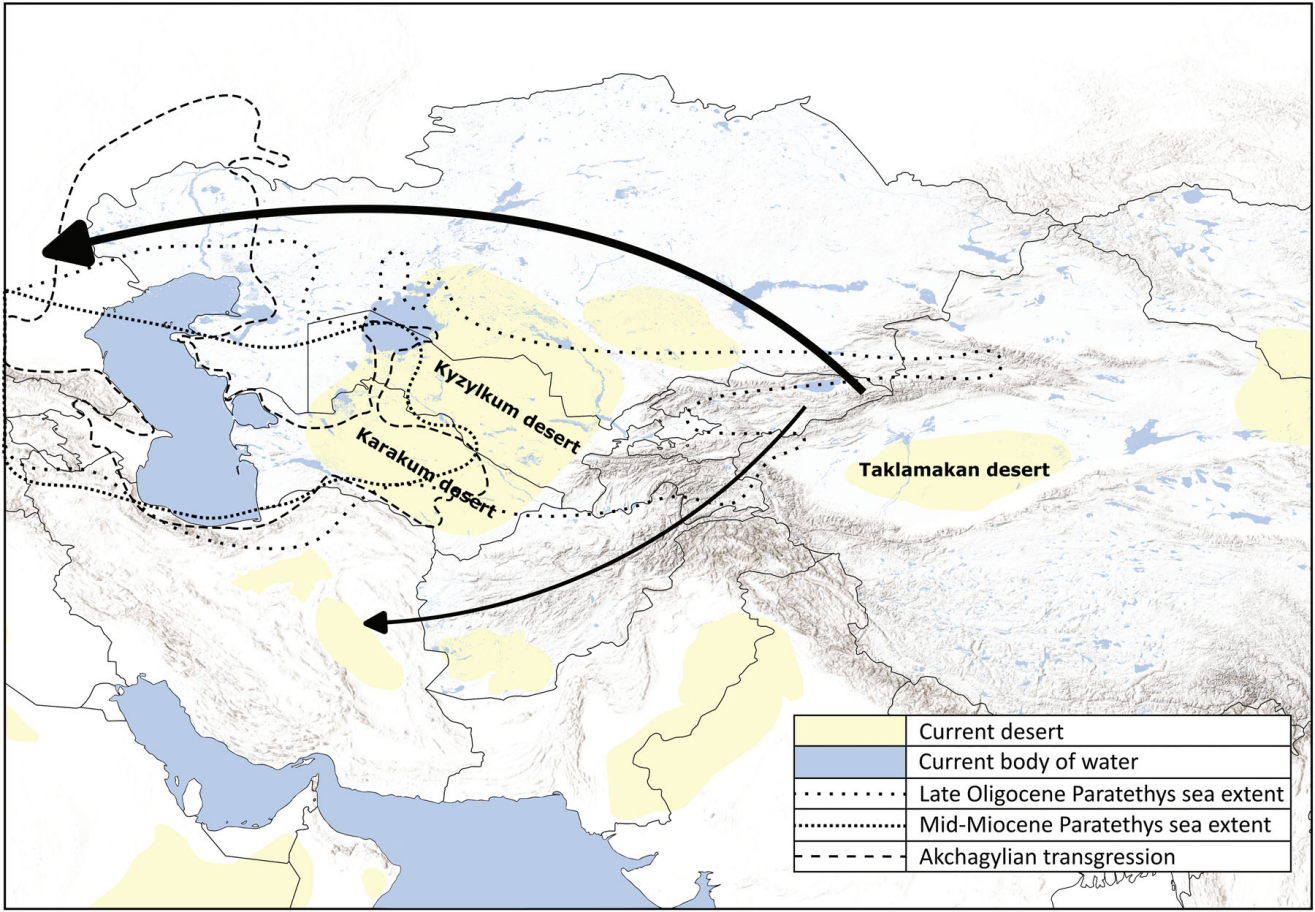
Map of the Asian area of the tulip distribution with present‐day deserts (Kelso and Patterson 2012), the estimated extent of the Parathys sea in the Oligocene (Li et al., 2020), the approximate extent of the Paratethys sea in the mid‐Miocene (Rögl, 1997), and the predicted extent of the Caspian Sea expansion during the Akchagylian transgression (Van Baak et al., 2013).

Our data suggest that the dispersal of tulips outside the ancestral Central Asian area has taken place several times between 7 and 3 Mya. The dispersal of tulips from this region occurred multiple times across the subgenera *Clusianae*, *Eriostemones*, and *Tulipa* (Figure 4). Despite multiple dispersal events, there appear to be only two distinct pathways out of Central Asia (Figure 5). Specifically, most of *Clusianae* and a small clade in subgenus *Tulipa* appear to have dispersed south from the Central Asian hotspot into Central and South Asia before diversifying, albeit leading to only a few species in both cases. In contrast, taxa that have led to large radiations in both the subgenera *Eriostemones* and *Tulipa* dispersed through the northwestern steppes of western Kazakhstan, southern Russia, and Ukraine, which were greatly expanding at the time (Hui et al., 2011; Hurka et al., 2019). This increased dispersal opportunities for tulips (Wesche et al., 2016), which eventually occupied the Turkey‐Iran‐Caucasus region. Our results indicate that the southern route presented more barriers to migration than the northern route, hence the limited dispersal of tulip diversity via this route. Specifically, although the Paratethys Sea in southern Central Asia greatly receded throughout the Oligocene into the Miocene (Dercourt et al., 1993; Rögl, 1997; Zhongshi et al., 2007; Popov et al., 2010), expansion events of the Caspian Sea in the late Pliocene‐early Pleistocene, especially the Akchagylian transgression, may have led to significant bodies of water acting as geographic barriers (Van Baak et al., 2013; Lazarev et al., 2021). In addition, the forming and expansion of the Karakum and Kyzylkum Deserts in the late Pliocene‐early Pleistocene era (~4–5 Mya) in areas where the Paratethys Sea had retreated (Atamuradov, 1994; Rustamov, 1994) presented further geographic barriers for dispersal into Iran, Anatolia and the Levant. The limited dispersal events of subgenera *Clusianae* and *Tulipa* via this southern route that did occur may have been through a circuitous route around the Karakum desert through the mountains of Afghanistan. This possibility is supported by the ranges of several current species from these clades, which encompass areas in southern Central Asia including in the mountains of Afghanistan and parts of Iran.

The diversity of tulips of subgenera *Eriostemones* and *Tulipa* that became established in the Turkey‐Iran‐Caucasus area remains unclear. However, tulip dispersal to the west is centered around species from the Saxatiles and Sylvestres clades of subgenus *Eriostemones* and the Tulipa clade of subgenus *Tulipa*. The separate diversifications of subgenera outside of Central Asia likely all arose from common ancestors of these clades that had a broad distribution covering parts of Central Asia, the northern grasslands, and the Turkey‐Iran‐Caucasus hotspot and in the case of subgenus *Tulipa* parts of the eastern Mediterranean as well. Several current species from these clades occupy extremely broad distributions, which may be remnants of ancestral taxa that bridged these regions. In *Eriostemones*, only a single clade links to the diversification of species outside Central Asia, which centers around a radiation in the Turkey‐Iran‐Caucasus region around 4 Mya, but led to migrations of taxa from this area of diversity to the Mediterranean and also eastward into Iran. In subgenus *Tulipa*, two radiations are evident, one in the Turkey‐Iran‐Caucasus region around 6 Mya, which also led to some species dispersing eastward into Iran where they now overlap with some species from subgenus *Clusianae*, and another in the Mediterranean around 2.5 Mya. The diversification of tulips of both subgenera in the Turkey‐Iran‐Caucasus region led to the creation of the secondary diversity center of the genus. The recent increase of tulip species in the Mediterranean, especially through polyploidy, has meant that certain researchers consider this region a new speciation center for the genus (Botschantzeva, 1982). Our work provides some evidence to support multiple recent migrations and speciation events in the region, but clearly the diversity of taxa in this region is far from that of the Turkey‐Iran‐Caucasus region or Central Asia and is of considerably smaller significance.

Broader Central Asia remains the most‐diverse region for tulips with the species of subgenera *Eduardoregelia* and *Orithyia* and the Kolpakowskianae, Multiflorae and Biflores clades, as well as many species of the Tulipa clade well represented in this ancestral area. Nonetheless, several individual species in the genus show range expansions from this region. Of note in Biflores is *Tulipa biflora*, which has dispersed from Central Asia into large parts of Eurasia within the last few million years, while most of its sister species remain native to Central Asia only. Furthermore, in the genus *Tulipa*, several semidesert‐dwelling species have evolved, which mostly occupy Central Asia, but some have distributions that extend into northern Turkmenistan and Afghanistan. Rarely, a species spread beyond the Karakum Desert, but several managed to do so, including *T. lehmanniana*. Here, we also found evidence of extensive dispersal of *T. undulatifolia*, but we note that its taxonomic position and distributional understanding are tenuous due to uncertainty in the sampled specimens, historical taxonomic issues, and poor records of occurrence. No biogeographical inferences have therefore been made based on our results for this species, and we highlight it as needing extensive further work. Overall, tulips appear to have dispersed out of Central Asia multiple times and through several different routes, but the steppe land of Kazakhstan, Russia, and the Caucasus has played a major role in the dispersal of tulips into what would become the secondary diversity hotspot of the Caucasus, Turkey, and Iran. From there, further dispersal created the range we know today stretching from western China to northern Africa and southern Iberia.

## CONCLUSIONS

Our research is timely given the need for an updated phylogenetic framework for the genus, especially because new species continue to be described based on morphology (e.g., De Groot and Zonneveld, 2022; Rukšāns and Zubov, 2022; Wilson et al., 2022, Moein et al., 2023; De Groot and Zonneveld, 2024; De Groot, 2024). We have developed phylogenies inferred from the largest sequence data sets to date, allowing us to not only investigate plastome structure in tulips and reassess elements of the taxonomy of this genus, but also carry out the first investigation into the evolutionary and biogeographic history of *Tulipa*. We have shown that plastome structure is consistent across the genus, and although species misidentification is somewhat common in the genus, we were able to make a number of taxonomic discoveries and insights. We formally described the new subgenus *Eduardoregelia* and from our work, we were able to propose where synonymization or reinstatement of species may be warranted providing a baseline for future taxonomic studies to resolve some of these issues.

We report that there is still significant work to be undertaken in this genus, especially surrounding species complexes. Our phylogenies highlight several areas that will require further extensive sampling and the use of large nuclear marker data sets to better understand evolutionary relationships, including how wild diversity connects to horticulture. Our work highlights *Tulipa scardica* and *T. suaveolens* as being of particular interest, alongside other species such as *T. agenensis*, *T. hungarica*, and tulip species in the Balkans, which warrant further investigation as well. Further work is also needed to assess species and synonyms for which we lacked material and for those specimens for which we had material but could not make concrete taxonomic suggestions. In addition, some species complexes may require population genetic techniques to unravel relationships and hybridization effects; in particular, we highlight here the mostly large red flowering tulips that constituted the former sections *Lanatae, Vinistriatae,* and *Spiranthera* as well as the clade Biflores. We emphasize that extensive collaborative efforts will be needed to keep the taxonomy of *Tulipa* up to date and that an integrated approach that uses morphology, genetics, and biogeography will be crucial.

Today, the distribution of native wild tulips covers large areas of Eurasia from north Africa and Spain across the Mediterranean, Middle East and Central Asia to western China (Everett, 2013; Christenhusz et al., 2013a; POWO, 2025). Yet, there remains one extremely significant diversity hotspot for tulips, Central Asia, where tulips can be found across alpine meadows, subalpine forests, alpine steppes, and desert steppes. Even though a secondary diversity hotspot across Turkey‐Caucasus‐Iran also exists, we have provided evidence to show that broader Central Asia is likely the area of origin of tulips and has been the most important region in the evolutionary history of this genus, with the Central Asian hotspot crucial in the diversification of most subgenera and clades. The climate and landscape of Central Asia have undergone many changes over the history of this genus with major orographic changes, persistent aridification, global cooling and increasing areas of grazed grassland and steppe playing a significant role in the speciation of tulips. We also showed multiple dispersal events of the genus out of Central Asia, with the steppes of Kazakhstan, Russia and the Caucasus, providing the primary pathway for dispersal of tulips westward.

Overall, we have presented an essential phylogenetic backbone for *Tulipa* and highlighted the importance of Central Asia in the evolutionary history of wild tulips. This reinforces the importance of phylogenetics as a key part of a stable taxonomy, which is in turn essential for progress in conservation (Mace, 2004; Garnett and Christidis, 2017) and reemphasizes the significance of the mountains of Central Asia biodiversity hotspot for global plant diversity (Critical Ecosystem Partnership Fund, 2016). This rugged landscape that harbors an array of wild fruit and nut trees (Cantarello et al., 2014; Orozumbekov, Cantarello and Newton, 2015; Wilson et al., 2019) and iconic megafauna such as snow leopards can now reliably be called the birthplace of tulips, with the conservation of its diverse ecosystems potentially promoted under this flagship flower (Pany and Heidinger, 2017).

## AUTHOR CONTRIBUTIONS

B.W. planned the work, collected the data, analyzed the data, wrote and edited the manuscript and created and revised the figures; M.J.M.C. helped plan the work, provided samples, interpreted the data, wrote and edited the manuscript; N.W.‐H. helped plan the work, analyzed and interpreted the data and edited the manuscript; N.B. helped collect samples; M.B. helped collect samples; C.C. helped interpret the data and edited the manuscript; D.D. helped collect samples; A.D. helped collect samples; M.F. helped collect samples; T.F. helped collect samples; S.d.G. helped collect samples; A.H. helped collect samples and edited the manuscript; G.A.L. helped plan the work and collected samples; K.T.S. helped plan the work and collected samples; O.S. helped collect samples and facilitated fieldwork; K.S.T. helped collect samples and edited the manuscript; B.J.M.Z. helped collect samples; S.F.B. planned and funded the work, interpreted the data, wrote and edited the manuscript and created and revised the figures.

## Supporting information


**Appendix S1:** Reference genomes used to extract plastid reads.

**Appendix S2:** The reference genomes used in the annotation process.

**Appendix S3**: List of plastome sequences from GenBank and their accession numbers.

**Appendix S4:** 35S rDNA sequences generated using *L. tsingtauense* reference.

**Appendix S5**: Full plastome phylogeny including identified erroneous specimens.

**Appendix S6:** The 80 specimens used to infer the species tree are listed.

**Appendix S7:** Information on the 245 specimens successfully sequenced for this study.

**Appendix S8:** Annotated genes across all *Tulipa* plastid assemblies.

**Appendix S9:** Phylogenetic relationships in the tribe *Tulipeae* inferred from plastome data.

**Appendix S10:** Phylogeny inferred using the partitioned 35S rDNA data set through a maximum likelihood approach.

**Appendix S11:** (a) Plastome (left) and 35S rDNA (right) phylogenies.

**Appendix S12**: Dated tree and lineage‐through‐time analyses.

**Appendix S13:** Geological time scale of epochs and ages relevant to the evolutionary history of the genus *Tulipa*.

## Data Availability

Raw sequence data have been uploaded to NCBI's Sequence Read Archive (SRA) under BioProject ID: PRJNA1017141. Assembled plastome data can be found in NCBI's GenBank under accession numbers OR288276–OR288520, and assembled 35S sequences have also been uploaded to NCBI's GenBank under accession numbers OR380721–OR380966. Specific details of each specimen's accession numbers can be found in Appendix 1.

## Appendix 1 Voucher information for taxa studied and GenBank accession information for plastome and 35S rDNA references. Heading “Named as” refers to the taxon assigned at time of collection.

**Table:**

Accepted taxon	Named as	Code	Collector and collection number if wild collected	Herbarium	Voucher no.	GenBank	
						Plastome	35S rDNA
*Tulipa biflora*	binutans	1	B. Wilson 1	CGG	20220345*H	OR288276	OR380722
*Tulipa lehmanniana*	zenaidae	2	B. Wilson 4	CGG	20220443*H	OR288277	OR380723
*Tulipa ferganica*	–	3	B. Wilson 6	CGG	20220363*H	OR288278	OR380724
*Tulipa talassica*	–	4	B. Wilson 7	CGG	20220405*H	OR288279	OR380725
*Tulipa tetraphylla*	–	5	B. Wilson 9	CGG	20220413*H	OR288280	OR380726
*Tulipa turkestanica*	–	6	B. Wilson 11	CGG	20220417*H	OR288281	OR380727
*Tulipa korolkowii*	rosea	7	B. Wilson 14	CGG	20220400*H	OR288282	OR380728
*Tulipa korolkowii*	–	8	B. Wilson 16	CGG	20220387*H	OR288283	OR380729
*Tulipa dasystemon*	–	9	B. Wilson 18	CGG	20220350*H	OR288284	OR380730
*Tulipa fosteriana*	affinis	10	B. Wilson 19	CGG	20220331*H	OR288285	OR380731
*Tulipa turkestanica*	–	13	B. Wilson 26	CGG	20220422*H	OR288286	OR380732
*Tulipa fosteriana*	affinis	14	B. Wilson 28	CGG	20220334*H	OR288287	OR380733
*Tulipa ferganica*	–	16	B. Wilson 31	CGG	20220364*H	OR288288	OR380734
*Tulipa platystemon*	–	17	B. Wilson 34	CGG	20220398*H	OR288289	OR380735
*Tulipa ferganica*	–	18	B. Wilson 35	CGG	20220366*H	OR288290	OR380736
*Tulipa urumiensis*	tarda	22	B. Wilson 43	CGG	20220408*H	OR288291	OR380737
*Tulipa* sp. unknown (Chuy)	–	23	B. Wilson 45	CGG	20220424*H	OR288292	OR380738
*Tulipa ostrowskiana*	–	27	B. Wilson 52	CGG	20220391*H	OR288293	OR380739
*Tulipa dasystemon*	–	31	B. Wilson 61	CGG	20220354*H	OR288294	OR380740
*Tulipa lehmanniana*	zenaidae	34	B. Wilson 66	CGG	20220445*H	OR288295	OR380741
*Tulipa greigii*	–	35	B. Wilson 67	CGG	20220371*H	OR288296	OR380742
*Tulipa tetraphylla*	–	39	B. Wilson 76	CGG	20220415*H	OR288297	OR380743
*Tulipa dasystemon*	dasystemonoides	40	B. Wilson 79	CGG	20220358*H	OR288298	OR380744
*Tulipa × tschimganica*	anadroma	41	B. Wilson 80	CGG	20220338*H	OR288299	OR380745
*Tulipa × tschimganica*	anadroma	42	B. Wilson 82	CGG	20220336*H	OR288300	OR380746
*Tulipa zonneveldii*	–	43	B. Wilson 84	CGG	20220448*H	OR288301	OR380747
*Tulipa zonneveldii*	–	44	B. Wilson 86	CGG	20220450*H	OR288302	OR380748
*Tulipa* sp. nov.	–	45	B. Wilson 88	CGG	20220389*H	OR288303	OR380749
*Tulipa dubia*	–	48	B. Wilson 93	CGG	20220361*H	OR288304	OR380750
*Tulipa bifloriformis*	–	49	B. Wilson 94	CGG	20220339*H	OR288305	OR380751
*Tulipa kaufmanniana*	–	50	B. Wilson 96	CGG	20220378*H	OR288306	OR380752
*Tulipa* sp. unknown (Chatkal)	–	51	B. Wilson 98	CGG	20220436*H	OR288307	OR380753
*Tulipa kaufmanniana*	–	52	B. Wilson 100	CGG	20220380*H	OR288308	OR380754
*Tulipa bifloriformis*	–	57	B. Wilson 110	CGG	20220343*H	OR288309	OR380755
*Tulipa greigii*	–	58	B. Wilson 112	CGG	20220374*H	OR288310	OR380756
*Tulipa* sp. unknown (Talas)	–	59	B. Wilson 115	CGG	20220441*H	OR288311	OR380757
*Tulipa ostrowskiana*	–	60	B. Wilson 116	CGG	20220395*H	OR288312	OR380758
*Tulipa greigii*	–	62	B. Wilson 118	CGG	20220376*H	OR288313	OR380759
*Tulipa biflora*	–	A	–	CGG	CGG17182	OR288314	OR380760
*Tulipa bifloriformis*	–	B	–	CGG	CGG17183	OR288315	OR380761
*Tulipa lehmanniana*	–	C	–	CGG	CGG17184	OR288335	OR380781
*Tulipa saxatilis*	–	D	–	CGG	CGG17186	OR288337	OR380783
*Tulipa saxatilis*	–	D2	–	CGG	14836	OR288338	OR380784
*Tulipa greigii*	–	E	–	CGG	CGG17185	OR288339	OR380785
*Tulipa greigii*	–	E2	–	K	001936030	OR288340	OR380786
*Tulipa montana*	–	F	–	CGG	CGG17180	OR288348	OR380794
*Tulipa kaufmanniana*	–	G	–	CGG	CGG17181	OR288349	OR380795
*Tulipa tetraphylla*	–	H	–	CGG	CGG17187	OR288361	OR380807
*Tulipa turkestanica*	–	I	–	CGG	CGG17179	OR288362	OR380808
*Tulipa dasystemon*	–	J	–	CGG	20080423*H	OR288363	OR380809
*Tulipa dasystemon*	–	K	–	CGG	20080418*H	OR288365	OR380811
*Tulipa vvedenskyi*	–	L	–	K	K001612283	OR288430	OR380876
*Tulipa vvedenskyi*	–	L2	–	K	K001612264	OR288431	OR380877
*Tulipa × tschimganica*	–	M	–	K	K001612288	OR288432	OR380878
*Tulipa ingens*	tubergeniana	N	–	K	K001767095	OR288433	OR380879
*Tulipa undulatifolia*	–	O	–	K	K001612279	OR288520	OR380966
*Tulipa ostrowskiana*	–	P	–	K	K001612267	OR288434	OR380880
*Tulipa biflora*	sogdiana	Q	–	K	001936029	OR288435	OR380881
*Tulipa orphanidea*	–	R	–	K	K001612296	OR288436	OR380882
*Tulipa humilis*	–	S	–	K	K001767091	OR288437	OR380883
*Tulipa clusiana* (f. *cashmeriana*)	–	T	–	K	2004‐3703	OR288438	OR380884
*Tulipa urumiensis*	tarda	U	–	K	K001612263	OR288466	OR380912
*Tulipa butkovii*	–	V	–		Not enough material to collect ‐ remains in living collection under 1978‐838 NYRK	OR288488	OR380934
*Tulipa lemmersii*	–	W	–	CGG	CGG17188	OR288489	OR380935
*Tulipa* cv. Duc van Tol	–	X	–	CGG	CGG17189	OR288490	OR380936
*Tulipa jacquesii*	–	Jac	G. Lazkov & K. Shalpykov	FRU	1904190019	OR288364	OR380810
*Tulipa foliosa*	armena var lycica	Kew1	–	K	K001612285	OR288366	OR380812
*Tulipa biflora*	binutans	Kew2	–	K	K001612274	OR288367	OR380813
*Tulipa carinata*	–	Kew4	–	K	K001767092	OR288368	OR380814
*Tulipa clusiana*	–	Kew5	–	K	K001767083	OR288369	OR380815
*Tulipa clusiana*	aitchisonii	Kew6	–	K	K001612282	OR288370	OR380816
*Tulipa dasystemon*	dasystemonoides	Kew7	–	K	K001612295	OR288371	OR380817
*Tulipa kaufmanniana*	–	Kew8	–	K	K001612291	OR288372	OR380818
*Tulipa montana*	chrysantha	Kew9	–	K	K001612290	OR288373	OR380819
*Tulipa orithyioides*	–	Kew10	–	K	K001936033	OR288374	OR380820
*Tulipa orphanidea*	hageri	Kew11	–	K	K001612294	OR288375	OR380821
*Tulipa orphanidea*	theophrastii	Kew12	–	K	K001612297	OR288376	OR380822
*Tulipa praestans*	–	Kew13	–	K	K001767087	OR288377	OR380823
*Tulipa saxatilis*	bakeri	Kew14	–	K	K001767086	OR288378	OR380824
*Tulipa sp*. unknown	–	Kew15	–	K	K001767085	OR288379	OR380825
*Tulipa sylvestris*	biebersteiniana	Kew16	–	K	K001612289	OR288380	OR380826
*Tulipa sylvestris*	patens	Kew17	–	K	K001612268	OR288381	OR380827
*Tulipa systola*	–	Kew18	–	K	K001612281	OR288382	OR380828
*Tulipa ×tschimganica*	–	Kew19	–	K	K001767088	OR288383	OR380829
*Tulipa turkestanica*	–	Kew20	–	K	K001936032	OR288384	OR380830
*Tulipa undulatifolia*	micheliana	Kew21	–	K	K001612287	OR288385	OR380831
*Tulipa borszczowii*	–	Kew22	–	Kew Living Collection and AA	MSB ID 0827005 (Kew living collection 20191973)	OR288386	OR380832
*Tulipa hungarica*	–	Kew23	–	K	K001612293	OR288387	OR380833
*Tulipa lemmersii*	–	Kew24	–	K	K001612269	OR288388	OR380834
*Tulipa hungarica*	rhodopea	Kew25	–	K	20191975	OR288389	OR380835
*Tulipa talassica*	–	Kew26	–	K	K001612286	OR288390	OR380836
*Tulipa agenensis*	–	Kew27	–	CGG	20220558*H (13353)	OR288391	OR380837
*Tulipa alberti*	–	Kew28	–	K	K001612262	OR288392	OR380838
*Tulipa altaica*	–	Kew29	–	Dead	was Kew living collection 2017288	OR288393	OR380839
*Tulipa cinnabarina*	–	Kew31	–	CGG	20220557*H (13364)	OR288394	OR380840
*Tulipa cretica*	–	Kew32	–	K (CGG)	K001767084	OR288395	OR380841
*Tulipa systola*	florenskyi	Kew33	–	K (CGG)	K001767093	OR288396	OR380842
*Tulipa hoogiana*	–	Kew34	–	K (CGG)	K001612275	OR288397	OR380843
*Tulipa hoogiana*	–	Kew35	–	K (CGG)	K001612276	OR288398	OR380844
*Tulipa ingens*	–	Kew36	–	K (CGG)	K001767089	OR288399	OR380845
*Tulipa jacquesii*	–	Kew37	–	K (CGG)	K001612270	OR288400	OR380846
*Tulipa kolpakowskiana*	–	Kew38	–	K (CGG)	K001612284	OR288401	OR380847
*Tulipa undulatifolia*	micheliana	Kew39	–	K	K001767090	OR288402	OR380848
*Tulipa montana*	–	Kew40	–	K (CGG)	K001612277	OR288403	OR380849
*Tulipa suaveolens*	schrenkii	Kew42	–	Dead	was Kew living collection 2009209	OR288404	OR380850
*Tulipa suaveolens*	schrenkii	Kew43	–	K	K003945291	OR288405	OR380851
*Tulipa sosnowskyi*	–	Kew44	–	K	K001612266	OR288406	OR380852
*Tulipa systola*	stapfi	Kew45	–	CGG	13358	OR288407	OR380853
*Tulipa sylvestris*	australis talijevii	Kew46	–	K (CGG)	K001612280	OR288408	OR380854
*Tulipa ulophylla*	–	Kew47	–	K	K001612292	OR288409	OR380855
*Tulipa uniflora*	–	Kew48	–	Dead	photo available ‐was Kew living collection 20042147	OR288410	OR380856
*Tulipa dasystemon*	neustruevae	Kew49	–	K (CGG)	K001612265	OR288411	OR380857
*Tulipa albanica*	–	Herb1	–	C	10014169	OR288503	OR380949
*Tulipa agenensis*	–	Edi1	–	CGE	CGE00080401	OR288341	OR380787
*Tulipa cretica*	–	Edi2	–	CGE	CGE00080402	OR288342	OR380788
*Tulipa × gesneriana*	–	Edi3	–	CGE	CGE00080897	OR288343	OR380789
*Tulipa lanata*	–	Edi4	–	CGE	CGE00080400	OR288344	OR380790
*Tulipa praestans*	–	Edi5	–	CGE	CGE00080899	OR288345	OR380791
*Tulipa heterophylla*	–	Edi6	–	CGE	CGE00080898	OR288346	OR380792
*Tulipa sprengeri*	–	Edi7	–	CGE	CGE00080403	OR288347	OR380793
*Tulipa praestans*	–	Tjk1	M. Boboev & S. Yoqubov 1	CGG	20230086*H	OR288439	OR380885
*Tulipa linifolia*	–	Tjk2	M. Boboev & S. Yoqubov 3	CGG	17541	OR288440	OR380886
*Tulipa linifolia*	–	Tjk3	M. Boboev & S. Yoqubov 5	CGG	17549	OR288441	OR380887
*Tulipa subquinquefolia*	–	Tjk4	M. Boboev & S. Yoqubov 7	CGG	20230083*H	OR288442	OR380888
*Tulipa anisophylla*	–	Tjk5	M. Boboev & S. Yoqubov 9	CGG	20230078*H	OR288443	OR380889
*Tulipa praestans*	subpraestans	Tjk6	M. Boboev & S. Yoqubov 11	CGG	17543	OR288444	OR380890
*Tulipa vvedenskyi*	–	Tjk7	M. Boboev & S. Yoqubov 13	CGG	20230085*H	OR288445	OR380891
*Tulipa ingens*	–	Tjk8	M. Boboev & S. Yoqubov 15	CGG	20230079*H	OR288446	OR380892
*Tulipa praestans*	–	Tjk9	M. Boboev & S. Yoqubov 17	CGG	17538	OR288447	OR380893
*Tulipa hissarica*	–	Tjk10	M. Boboev & S. Yoqubov 19	CGG	17545	OR288448	OR380894
*Tulipa anisophylla*	korshinskyi	Tjk11	M. Boboev & S. Yoqubov 21	CGG	BM21	OR288449	OR380895
*Tulipa scardica*	–	Herb2	–	B	101068479	OR288504	OR380950
*Tulipa albanica*	–	Bal1	A. Hajdari & B. Pulaj 1	UP	00000158	OR288316	OR380762
*Tulipa albanica*	–	Bal2	A. Hajdari & B. Pulaj 2	UP	00000157	OR288317	OR380763
*Tulipa albanica*	–	Bal3	A. Hajdari & B. Pulaj 3	UP	00000156	OR288318	OR380764
*Tulipa kosovarica*	–	Bal4	A. Hajdari & B. Pulaj 4	UP	00000155	OR288319	OR380765
*Tulipa kosovarica*	–	Bal5	A. Hajdari & B. Pulaj 5	UP	00000154	OR288320	OR380766
*Tulipa kosovarica*	–	Bal6	A. Hajdari & B. Pulaj 6	UP	00000153	OR288321	OR380767
*Tulipa kosovarica*	–	Bal7	A. Hajdari & B. Pulaj 7	UP	00000152	OR288322	OR380768
*Tulipa* sp. nov.	–	Bal8	A. Hajdari & B. Pulaj 8	UP	00000150	OR288323	OR380769
*Tulipa luanica*	–	Bal9	A. Hajdari & B. Pulaj 9	UP	00000149	OR288324	OR380770
*Tulipa luanica*	–	Bal10	A. Hajdari & B. Pulaj 10	UP	00000146	OR288325	OR380771
*Tulipa luanica*	–	Bal11	A. Hajdari & B. Pulaj 11	UP	00000148	OR288326	OR380772
*Tulipa luanica*	–	Bal12	A. Hajdari & B. Pulaj 12	UP	00000145	OR288327	OR380773
*Tulipa scardica*	–	Bal13	A. Hajdari & B. Pulaj 13	UP	00000167	OR288328	OR380774
*Tulipa scardica*	–	Bal14	A. Hajdari & B. Pulaj 14	UP	00000166	OR288329	OR380775
*Tulipa serbica*	–	Bal15	A. Hajdari & B. Pulaj 15	UP	00000164	OR288330	OR380776
*Tulipa serbica*	–	Bal16	A. Hajdari & B. Pulaj 16	UP	00000163	OR288331	OR380777
*Tulipa sylvestris* subsp. *sylvestris*	–	Bal17	A. Hajdari & B. Pulaj 17	UP	00000161	OR288332	OR380778
*Tulipa sylvestris* subsp*. australis*	–	Bal18	A. Hajdari & B. Pulaj 18	UP	00000160	OR288333	OR380779
*Tulipa sylvestris* subsp*. sylvestris*	–	Bal19	A. Hajdari & B. Pulaj 19	UP	00000159	OR288334	OR380780
*Tulipa heterophylla*	–	Kyr1	G. Lazkov & K. Shalpykov	FRU	2105200001	OR288412	OR380858
*Tulipa heterophylla*	–	Kyr2	G. Lazkov & K. Shalpykov	FRU	0406200002	OR288413	OR380859
*Tulipa heterophylla*	–	Kyr3	G. Lazkov & K. Shalpykov	FRU	0306200003	OR288414	OR380860
*Tulipa heterophylla*	–	Kyr4	G. Lazkov & K. Shalpykov	FRU	0206200004	OR288415	OR380861
*Tulipa kolpakowskiana*	–	Kyr5	G. Lazkov & K. Shalpykov	FRU	2703200005	OR288416	OR380862
*Tulipa talassica*	–	Kyr6	G. Lazkov & K. Shalpykov	FRU	2604200006	OR288417	OR380863
*Tulipa jacquesii*	–	Kyr7	G. Lazkov & K. Shalpykov	FRU	2103200007	OR288418	OR380864
*Tulipa koyuncui*	–	Tur1	M. Fırat 35497	VANF	16281	OR288454	OR380900
*Tulipa biflora*	–	Tur2	M. Fırat 35480	VANF	16283	OR288455	OR380901
*Tulipa biflora*	–	Tur3	M. Fırat 35494	VANF	16277	OR288456	OR380902
*Tulipa* sp. unknown	–	Tur4	M. Fırat 35484	VANF	16288	OR288457	OR380903
*Tulipa koyuncui*	–	Tur5	M. Fırat 35488	VANF	16289	OR288458	OR380904
*Tulipa humilis*	–	Tur6	M. Fırat 35500	VANF	16293	OR288459	OR380905
*Tulipa biflora*	–	Tur7	M. Fırat 35481	VANF	16284	OR288460	OR380906
*Tulipa biflora*	–	Tur8	M. Fırat 35482	VANF	16285	OR288461	OR380907
*Tulipa* sp. unknown	–	Tur9	M. Fırat 35485	VANF	16287	OR288462	OR380908
*Tulipa* sp. unknown	–	Tur10	M. Fırat 35486	VANF	16286	OR288463	OR380909
*Tulipa koyuncui*	–	Tur11	M. Fırat 35489	VANF	16290	OR288464	OR380910
*Tulipa koyuncui*	–	Tur12	M. Fırat 35490	VANF	16291	OR288465	OR380911
*Tulipa lemmersii*	–	Zonn3	–	L	0822655	OR288491	OR380937
*Tulipa kolbintsevii*	–	Zonn4	–	L	0821329	OR288492	OR380938
*Tulipa zonneveldii*	–	Zonn5	–	L	3971774	OR288493	OR380939
*Tulipa jacquesii*	–	Zonn6	–	CGG	CGG18399	OR288494	OR380940
*Tulipa annae*	–	Zonn7	–	L	3986814	OR288495	OR380941
*Tulipa ivasczenkoae*	–	Zonn8	–	CGG	CGG18398	OR288496	OR380942
*Tulipa regelii*	–	Zonn9	–	CGG	CGG18400	OR288497	OR380943
*Tulipa uzbekistanica*	–	Zonn10	–	CGG	CGG18401	OR288498	OR380944
*Tulipa heteropetala*	–	Zonn11	–		L4513068	OR288499	OR380945
*Tulipa alberti*	–	Zonn12	–	CGG	CGG18402	OR288500	OR380946
*Tulipa alberti*	–	Zonn13	–	CGG	18465	OR288501	OR380947
*Tulipa dianaeverettiae*	–	Zonn14	–	L	L 3986813	OR288502	OR380948
*Tulipa heweri*	–	Chr 1	–	PG	M.J.M. Christenhusz no 9119	OR288335	OR380782
*Tulipa julia*	–	Herb4	–	E	EOO332503 2275*4	OR288505	OR380951
*Tulipa kuschkensis*	–	Herb5	–	E	EOO332529 2275*7	OR288506	OR380952
*Tulipa lehmanniana*	–	Herb6	–	E	EOO332532 2275*9	OR288507	OR380953
*Tulipa × gesneriana*	–	Herb7	–	E	EOO329775 2275*11	OR288508	OR380954
*Tulipa banuensis*	–	Herb8	–	E	EOO329548 2275*22	OR288509	OR380955
*Tulipa lehmanniana*	–	Herb9	–	E	EOO332534 2275*10	OR288510	OR380956
*Tulipa sosnowskyi*	–	Herb10	–	E	EOO329791 2275*27	OR288511	OR380957
*Tulipa banuensis*	–	Herb11	–	E	EOO373862 2275*23	OR288512	OR380958
*Tulipa harazensis*	–	Herb14	–	E	EOO329590 2275*13	OR288513	OR380959
*Tulipa hoogiana*	–	Herb15	–	E	EOO329594 2275*14	OR288514	OR380960
*Tulipa kuschkensis*	–	Herb16	–	E	EOO332528 2275*8	OR288515	OR380961
*Tulipa agenensis*	–	Herb17	–	E	EOO329512 2275*16	OR288516	OR380962
*Tulipa armena*	–	Herb18	–	E	EOO329523 2275*21	OR288517	OR380963
*Tulipa sosnowskyi*	–	Herb19	–	E	EOO329790 2275*28	OR288518	OR380964
*Tulipa armena*	–	Herb20	–	E	EOO329519 2275*20	OR288519	OR380965
*Tulipa cinnabarina*	–	Got1		GB	GB‐0219115	OR288350	OR380796
*Tulipa julia*		Got2	–	GB	GB‐0219118	OR288351	OR380797
*Tulipa suaveolens*	schrenkii	Got3	–	GB	GB‐0219121	OR288352	OR380798
*Tulipa armena*	–	Got4	–	GB	GB‐0219114	OR288353	OR380799
*Tulipa altaica*	–	Got5	–	GB	GB‐0219113	OR288354	OR380800
*Tulipa uniflora*	–	Got6	–	GB	GB‐0219122	OR288355	OR380801
*Tulipa regelii*	–	Got7	–	GB	GB‐0219119	OR288356	OR380802
*Tulipa kolbintsevii*	–	Got8		GB	GB‐0219112	OR288357	OR380803
*Tulipa heteropetala*	–	Got9	–	GB	GB‐0219116	OR288358	OR380804
*Tulipa heteropetala*	–	Got10	–	GB	GB‐0219117	OR288359	OR380805
*Tulipa regelii*	–	Got11	–	GB	GB‐0219120	OR288360	OR380806
*Tulipa uzbekistanica*	–	Uzb1	D. Dekhkonov & M Makhmudjanov 1	TASH	DD01042021002	OR288467	OR380913
*Tulipa × tschimganica*	–	Uzb2	D. Dekhkonov & E. Ortikov 1	TASH	DE12052021007	OR288468	OR380914
*Tulipa orithyioides*	–	Uzb3	O. Turginov & A. Rakhmatov 1	TASH	OA11062021003	OR288469	OR380915
*Tulipa greigii*	–	Uzb4	D. Dekhkonov, Z. Yusupov & M Makhmudjanov 1	TASH	DZD30042021006	OR288470	OR380916
*Tulipa vvedenskyi*	–	Uzb5	D. Dekhkonov 1	TASH	DD02042021001‐2	OR288471	OR380917
*Tulipa carinata*	–	Uzb6	O. Turginov, K. Obidjon & S. Pulatov 1	TASH	OO31032021023‐1	OR288472	OR380918
*Tulipa fosteriana*	–	Uzb7	U. Kadirov 1	TASH	UK16042021005	OR288473	OR380919
*Tulipa korolkowii*	–	Uzb8	D. Dekhkonov & M Makhmudjanov 2	TASH	DD01042021004‐1	OR288474	OR380920
*Tulipa lanata*	–	Uzb9	D. Dekhkonov, M Makhmudjanov & S. Pulatov 1	TASH	DD525032021022	OR288475	OR380921
*Tulipa ingens*	–	Uzb10	D. Dekhkonov & M Makhmudjanov 3	TASH	DD31032021027‐1	OR288476	OR380922
*Tulipa biflora*	–	Uzb11	K. Shomurodov, B Adilov & O. Abduraimov 1	TASH	53‐1	OR288477	OR380923
*Tulipa kaufmanniana*	–	Uzb12	D. Dekhkonov, Z. Yusupov & M Makhmudjanov 1	TASH	DZD30042021004	OR288478	OR380924
*Tulipa dubia*	–	Uzb13	N. Beshko & U Kadirov 1	TASH	1606202103	OR288479	OR380925
*Tulipa turkestanica*	–	Uzb14	K. Tojibaev, A. Batashov, F. Karimov & D. Dekhkonov 1	TASH	220320204	OR288480	OR380926
*Tulipa lehmanniana*	–	Uzb15	D. Dekhkonov & M Makhmudjanov 4	TASH	DD15042021011	OR288481	OR380927
*Tulipa talassica*	–	Uzb16	D. Dekhkonov 2	TASH	DD19042021002	OR288482	OR380928
*Tulipa scharipovii*	–	Uzb17	K. Tojibaev, A. Batashov, D. Dekhkonov & N. Khoshimov 1	TASH	20032020044	OR288483	OR380929
*Tulipa intermedia*	–	Uzb18	K. Tojibaev, A. Batashov, D. Dekhkonov & N. Khoshimov 2	TASH	2003202047‐5	OR288484	OR380930
*Tulipa ferganica*	–	Uzb19	K. Tojibaev, A. Batashov, F. Karimov & D. Dekhkonov 1	TASH	220320202‐2	OR288485	OR380931
*Tulipa butkovii*	–	Uzb20	D. Dekhkonov, Z. Yusupov & M Makhmudjanov 2	TASH	DZD30042021001	OR288486	OR380932
*Tulipa bifloriformis*	–	Uzb21	–	TASH	422003	OR288487	OR380933
*Tulipa lanata*	–	Tjk12	M. Boboev & S. Yoqubov 23	CGG	17547	OR288450	OR380896
*Tulipa korolkowii*	nitida	Tjk13	M. Boboev & S. Yoqubov 25	CGG	17550	OR288451	OR380897
*Tulipa linifolia*	maximowiczii	Tjk14	M. Boboev & S. Yoqubov 27	CGG	17539	OR288452	OR380898
*Tulipa korolkowii*	rosea	Tjk15	M. Boboev & S. Yoqubov 29	CGG	20230077*H	OR288453	OR380899
*Tulipa platystemon*	–	Kyr8	G. Lazkov & K. Shalpykov	FRU	0505210008	OR288419	OR380865
*Tulipa dubia*	–	Kyr9	G. Lazkov & K. Shalpykov	FRU	0605210009	OR288420	OR380866
*Tulipa korolkowii*	–	Kyr10	G. Lazkov & K. Shalpykov	FRU	1704210010	OR288421	OR380867
*Tulipa tetraphylla*	brachystemon	Kyr11	G. Lazkov & K. Shalpykov	FRU	2704210011	OR288422	OR380868
*Tulipa lehmanniana*	zenaidae	Kyr12	G. Lazkov & K. Shalpykov	FRU	2704210012	OR288423	OR380869
*Tulipa talassica*	–	Kyr13	G. Lazkov & K. Shalpykov	FRU	0805210013	OR288424	OR380870
*Tulipa talassica*	–	Kyr14	G. Lazkov & K. Shalpykov	FRU	0405210014	OR288425	OR380871
*Tulipa* sp. unknown	–	Kyr15	G. Lazkov & K. Shalpykov	FRU	0605210015	OR288426	OR380872
*Tulipa dasystemon*	–	Kyr16	G. Lazkov & K. Shalpykov	FRU	0805210016	OR288427	OR380873
*Tulipa dasystemon*	dasystemonoides	Kyr17	G. Lazkov & K. Shalpykov	FRU	0705210017	OR288428	OR380874
*Tulipa* sp. unknown	–	Kyr18	G. Lazkov & K. Shalpykov	FRU	0605210018	OR288429	OR380875
*Amana baohuaensis*	–	SRR12599520	–	–	–	–	OR380721
