# Supplementary material for: Phylogenomic insights into the genus Tulipa (Liliaceae): Taxonomy, evolution, and biogeography
Source: Am J Bot. 2026 Aug 4;113(8):e70232. doi: 10.1002/ajb2.70232 (PMC13496073; doi:10.1002/ajb2.70232)
Supplement: Supplementary file 1 [file AJB2-113-e70232-s001.pdf]

Appendix S4. 35S rDNA sequences generated using *L. tsingtauense* reference

Species	Subgenus	Sequence length
Tulipa biflora (binutans)	Eriostemones	5852
Tulipa linifolia	Clusianae	5852
Tulipa korolkowii	Tulipa	5856
Tulipa greigii	Tulipa	5856
Tulipa heterophylla	Orithyia	5858
