# Supplementary material for: Phylogenomic insights into the genus Tulipa (Liliaceae): Taxonomy, evolution, and biogeography
Source: Am J Bot. 2026 Aug 4;113(8):e70232. doi: 10.1002/ajb2.70232 (PMC13496073; doi:10.1002/ajb2.70232)
Supplement: Supplementary file 2 [file AJB2-113-e70232-s002.pdf]

Appendix S10. Phylogeny inferred using the partitioned 35S rDNA data set through a maximum likelihood approach. Tips that were believed to be errors were pruned, and the tree was rooted using a single specimen from Amana. Nonparametric bootstrap values are shown for branches. Tips are color-coded based on certain taxonomic processes or decisions linked to these specimens.

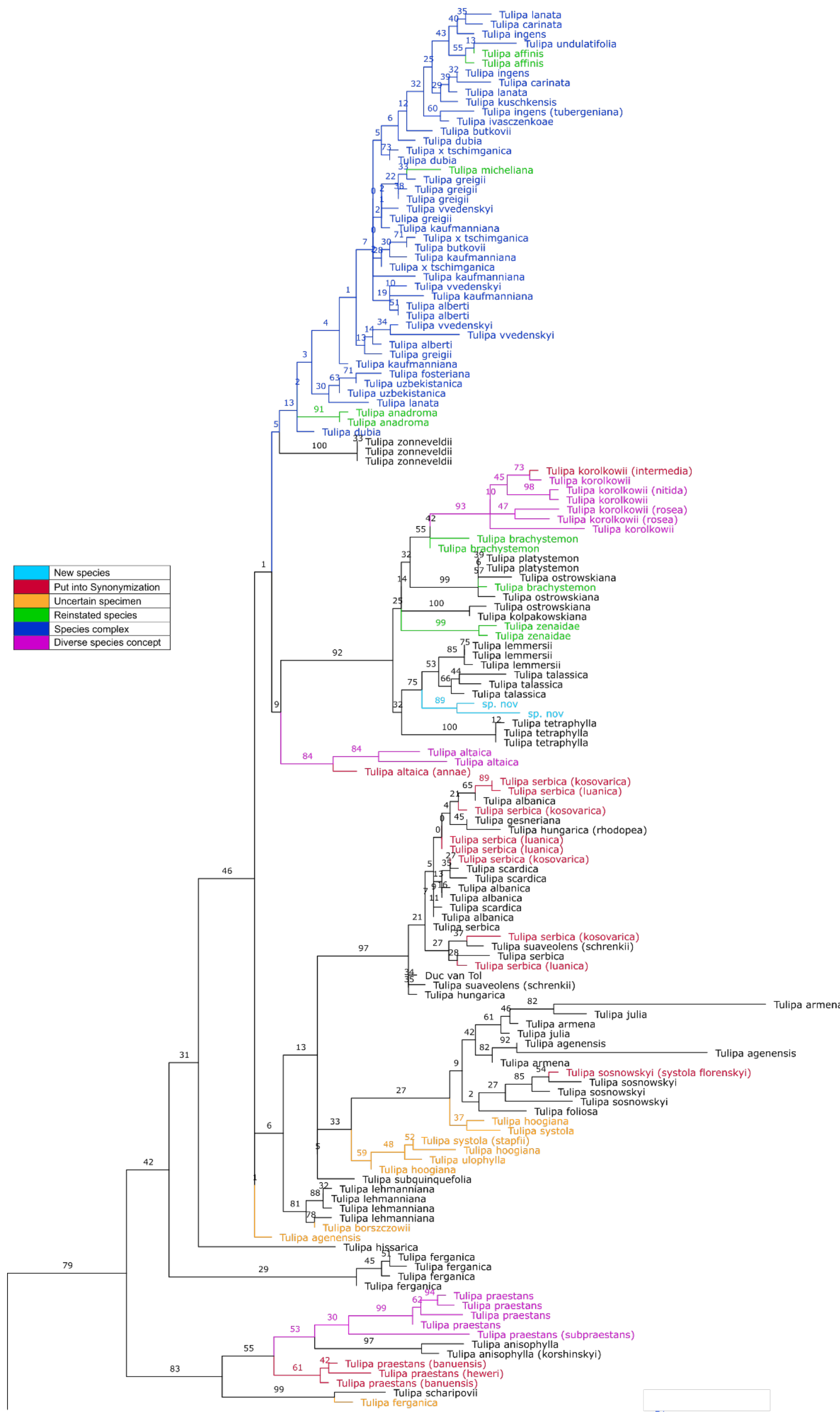

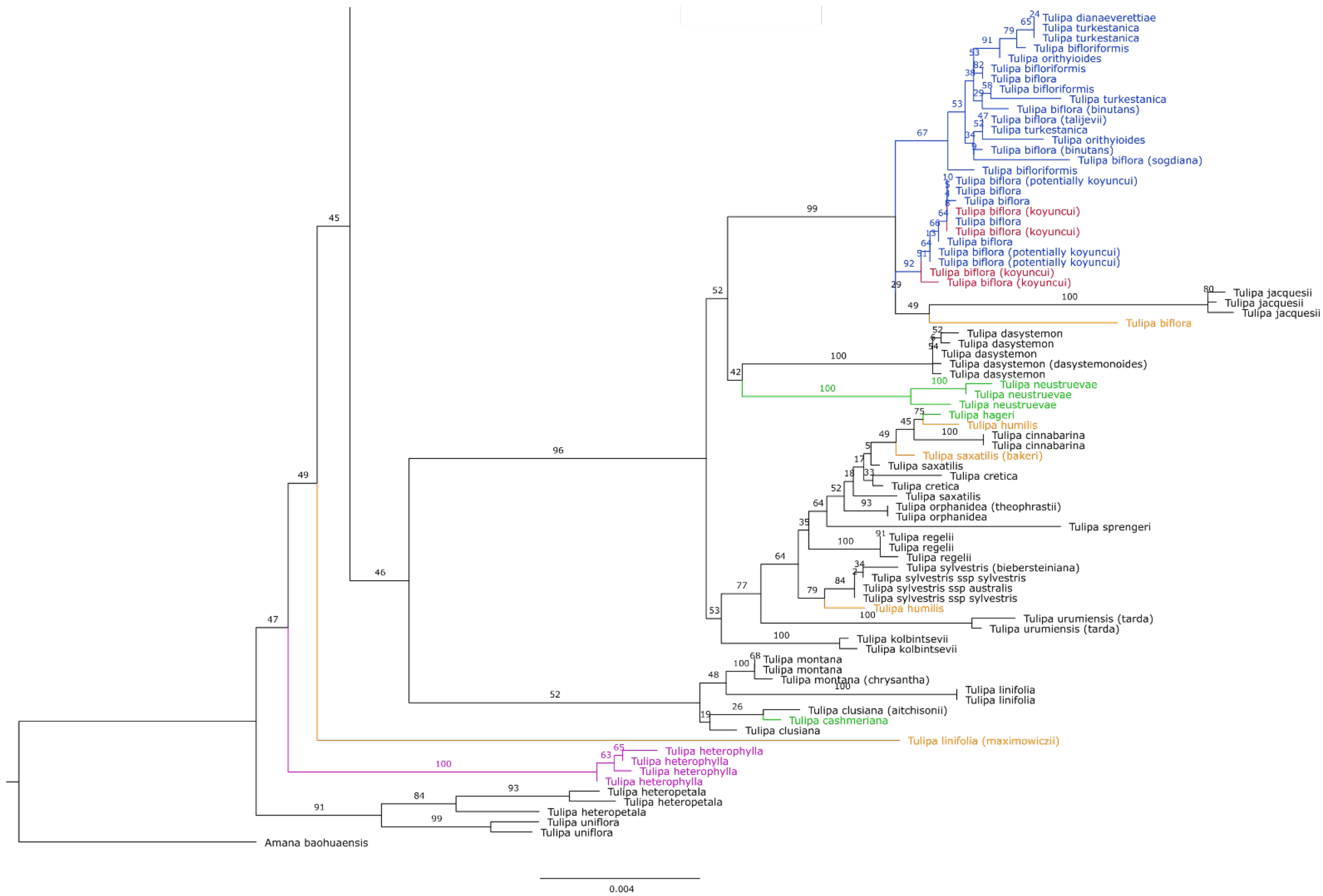

Tulipa dianaeverettiae
Tulipa turkestanica
Tulipa turkestanica
Tulipa bifloriformis
Tulipa orithyioides
Tulipa bifloriformis
Tulipa biflora
Tulipa bifloriformis
Tulipa turkestanica
Tulipa biflora (binutans)
Tulipa biflora (talijevii)
Tulipa turkestanica
Tulipa orithyioides
Tulipa biflora (binutans)
Tulipa biflora (sogdiana)
Tulipa bifloriformis
Tulipa biflora (potentially koyuncui)
Tulipa biflora
Tulipa biflora
Tulipa biflora (koyuncui)
Tulipa biflora
Tulipa biflora (koyuncui)
Tulipa biflora
Tulipa biflora (potentially koyuncui)
Tulipa biflora (potentially koyuncui)
Tulipa biflora (koyuncui)
Tulipa biflora (koyuncui)
Tulipa jacquesii
Tulipa jacquesii
Tulipa jacquesii
Tulipa biflora
Tulipa dasystemon
Tulipa dasystemon
Tulipa dasystemon
Tulipa dasystemon (dasystemonoides)
Tulipa dasystemon
Tulipa neustruevae
Tulipa neustruevae
Tulipa neustruevae
Tulipa hageri
Tulipa humilis
Tulipa cinnabarina
Tulipa cinnabarina
Tulipa saxatilis (bakeri)
Tulipa saxatilis
Tulipa cretica
Tulipa cretica
Tulipa saxatilis
Tulipa orphanidea (theophrastii)
Tulipa orphanidea
Tulipa sprengeri
Tulipa regelii
Tulipa regelii
Tulipa regelii
Tulipa sylvestris (biebersteiniana)
Tulipa sylvestris ssp sylvestris
Tulipa sylvestris ssp australis
Tulipa sylvestris ssp sylvestris
Tulipa humilis
Tulipa urumiensis (tarda)
Tulipa urumiensis (tarda)
Tulipa kolbintsevii
Tulipa kolbintsevii
Tulipa montana
Tulipa montana
Tulipa montana (chrysantha)
Tulipa linifolia
Tulipa linifolia
Tulipa clusiana (aitchisonii)
Tulipa cashmeriana
Tulipa clusiana
Tulipa linifolia (maximowiczii)
Tulipa heterophylla
Tulipa heterophylla
Tulipa heterophylla
Tulipa heterophylla
Tulipa heteropetala
Tulipa heteropetala
Tulipa heteropetala
Tulipa uniflora
Tulipa uniflora
Amana baohuaensis
0.004
