# Supplementary material for: Phylogenomic insights into the genus Tulipa (Liliaceae): Taxonomy, evolution, and biogeography
Source: Am J Bot. 2026 Aug 4;113(8):e70232. doi: 10.1002/ajb2.70232 (PMC13496073; doi:10.1002/ajb2.70232)
Supplement: Supplementary file 3 [file AJB2-113-e70232-s003.pdf]

Appendix S2. The reference genomes used in the annotation process

Species	NCBI Reference Sequence
Tulipa altaica	NC_044780
Tulipa sylvestris	MT261172
Erythronium sibiricum	NC_035681
Amana erythronioides	KY401424
Amana edulis	NC_034707
