# Supplementary material for: Phylogenomic insights into the genus Tulipa (Liliaceae): Taxonomy, evolution, and biogeography
Source: Am J Bot. 2026 Aug 4;113(8):e70232. doi: 10.1002/ajb2.70232 (PMC13496073; doi:10.1002/ajb2.70232)

Appendix S5. Full plastome phylogeny including identified erroneous specimens. The tree was inferred using partitioned plastome data under a maximum likelihood approach. Tips corresponding to putative errors are highlighted in red; those for specimens that could not be assigned to a species concept are in orange. The tree was rooted using two Gagea, five Amana, and two Erythronium specimens, but only the Amana and Erythronium outgroups are shown here. Nonparametric bootstrap support values are indicated for branches.

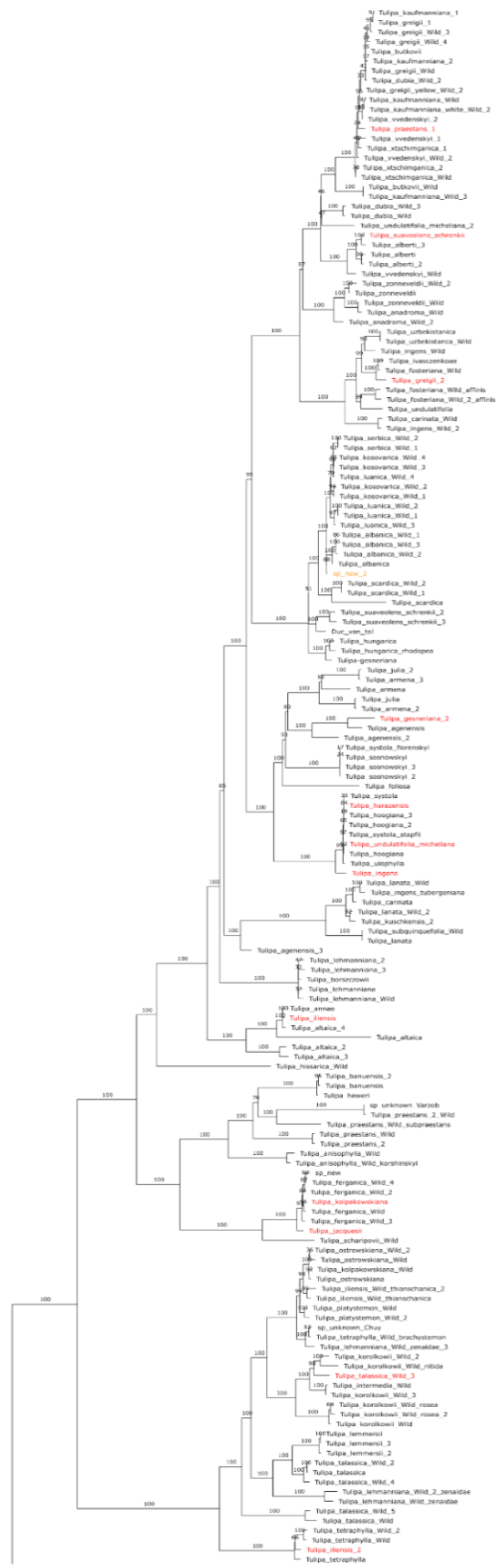

Continued on page below

Continued on page above

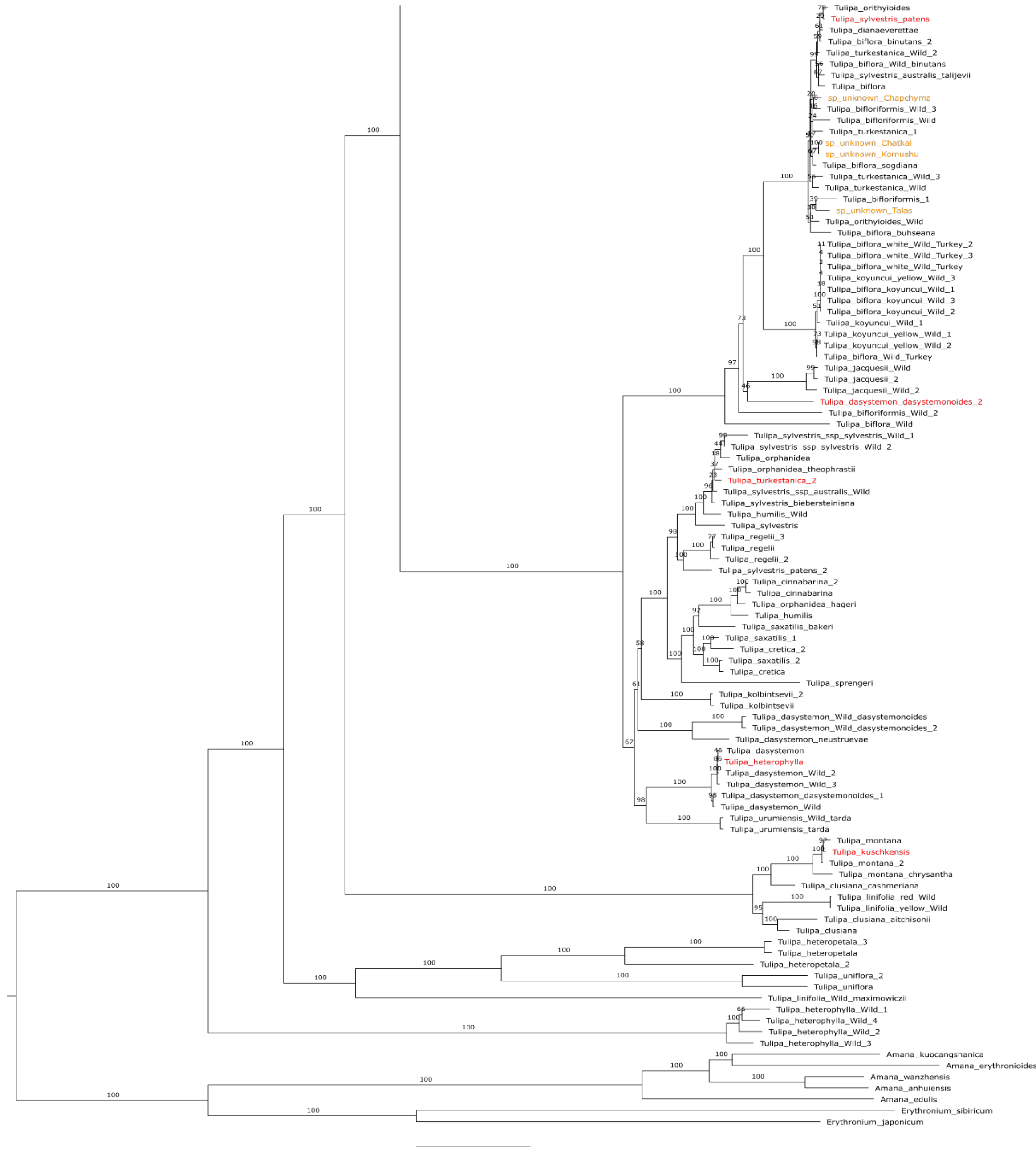

Supplement: Supplementary file 4 [file AJB2-113-e70232-s004.pdf]
