# Supplementary material for: Phylogenomic insights into the genus Tulipa (Liliaceae): Taxonomy, evolution, and biogeography
Source: Am J Bot. 2026 Aug 4;113(8):e70232. doi: 10.1002/ajb2.70232 (PMC13496073; doi:10.1002/ajb2.70232)
Supplement: Supplementary file 5 [file AJB2-113-e70232-s005.pdf]

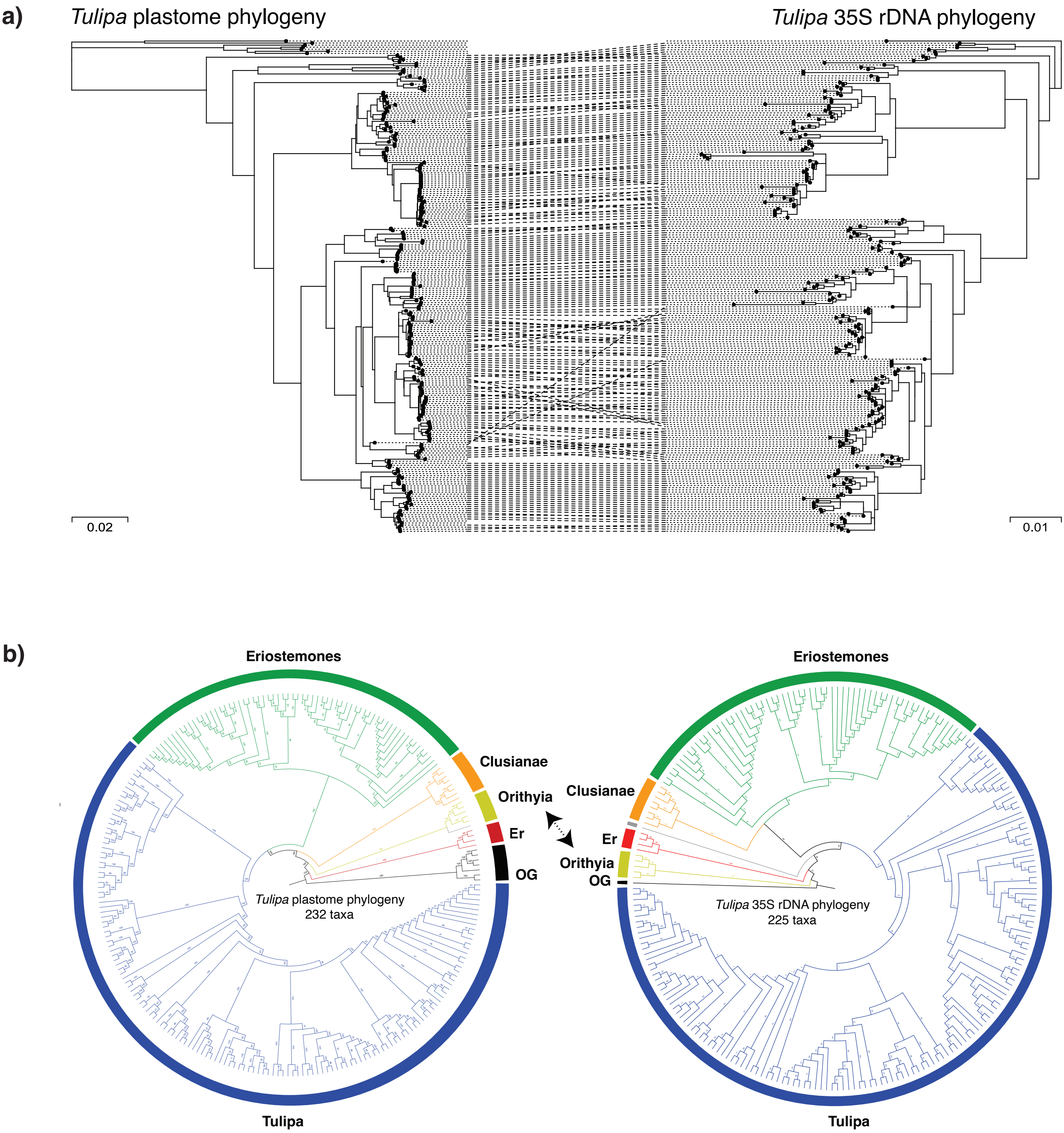

a)
Tulipa plastome phylogeny
Tulipa 35S rDNA phylogeny
0.02
0.01
b)
Eriostemones
Clusianae
Orithyia
Er
OG
Tulipa plastome phylogeny
232 taxa
Tulipa
Eriostemones
Clusianae
Er
Orithyia
OG
Tulipa 35S rDNA phylogeny
225 taxa
Tulipa
