# Supplementary material for: Phylogenomic insights into the genus Tulipa (Liliaceae): Taxonomy, evolution, and biogeography
Source: Am J Bot. 2026 Aug 4;113(8):e70232. doi: 10.1002/ajb2.70232 (PMC13496073; doi:10.1002/ajb2.70232)
Supplement: Supplementary file 6 [file AJB2-113-e70232-s006.pdf]

Appendix S1. Reference genomes used to extract plastid chloroplast reads.

**Species**	**NCBI reference sequence**
Amana anhuiensis	NC_034706
Amana edulis	NC_034707
Amana erythronioides	KY401424
Amana kuocangshanica	NC_034708
Amana wanzhensis	NC_034705
Erythronium sibiricum	NC_035681
