# Supplementary material for: Phylogenomic insights into the genus Tulipa (Liliaceae): Taxonomy, evolution, and biogeography
Source: Am J Bot. 2026 Aug 4;113(8):e70232. doi: 10.1002/ajb2.70232 (PMC13496073; doi:10.1002/ajb2.70232)
Supplement: Supplementary file 7 [file AJB2-113-e70232-s007.pdf]

Appendix S3. List of plastome sequences from GenBank and accession numbers.

Species	NCBI Reference Sequence
Tulipa sylvestris	MT261172
Tulipa biflora (buhseana)	MT316022
Tulipa iliensis	MT316023
Tulipa iliensis (thianschanica)	MT327741
Tulipa iliensis (thianschanica)	MW077738
Tulipa iliensis	MW077740
Tulipa sylvestris (patens)	MW077739
Tulipa altaica	MW077741
Tulipa altaica	NC_044780
Amana edulis	NC_034707
Amana anhuiensis	NC_034706
Amana kuocangshanica	NC_034708
Amana erythronioides	KY401424
Amana wanzhensis	NC_0347065
Erythronium japonicum	MT261155
Erythronium sibiricum	NC_035681
Gagea triflora	MT261157
Gagea (Lloydia) tibetica*	NC_058295
