# Supplementary material for: Phylogenomic insights into the genus Tulipa (Liliaceae): Taxonomy, evolution, and biogeography
Source: Am J Bot. 2026 Aug 4;113(8):e70232. doi: 10.1002/ajb2.70232 (PMC13496073; doi:10.1002/ajb2.70232)
Supplement: Supplementary file 8 [file AJB2-113-e70232-s008.pdf]

Appendix S6. The 80 specimens used to infer the species tree

Code	Original species name	Tip on species tree
**D**	*T. saxatilis*	*T. saxatilis*
**F**	*T. montana*	*T. montana*
**O**	*T. undulatifolia*	*T. undulatifolia*
**R**	*T. orphanidea*	*T. orphanidea*
**T**	*T. clusiana (cashmeriana)*	*T. cashmeriana*
**W**	*T. lemmersii*	*T. lemmersii*
**X**	Duc van Tol	Duc van Tol
**Edi1**	*T. agenensis*	*T. agenensis*
**Edi2**	*T. cretica*	*T. cretica*
**Edi7**	*T. sprengeri*	*T. sprengeri*
**Kew1**	*T. foliosa*	*T. foliosa*
**Kew4**	*T. carinata*	*T. carinata*
**Kew5**	*T. clusiana*	*T. clusiana*
**Kew11**	*T. orphanidea* (*hageri*)	*T. hageri*
**Kew18**	*T. systola*	*T. systola*
**Kew22**	*T. borszczowii*	*T. borszczowii*
**Kew23**	*T. hungarica*	*T. hungarica*
**Kew28**	*T. alberti*	*T. alberti*
**Kew29**	*T. altaica*	*T. altaica*
**Kew31**	*T. cinnabarina*	*T. cinnabarina*
**Kew34**	*T. hoogiana*	*T. hoogiana*
**Kew39**	*T. undulatifolia* (*micheliana*)	*T. micheliana*
**Kew43**	*T. suaveolens* (*schrenkii*)	*T. suaveolens*
**Kew44**	*T. sosnowskyi*	*T. sosnowskyi*
**Kew47**	*T. ulophylla*	*T. ulophylla*
**2**	*T. lehmanniana* (*zenaidae*)	*T. zenaidae*
**3**	*T. ferganica*	*T. ferganica*
**4**	*T. talassica*	*T. toktogulica*
**5**	*T. tetraphylla*	*T. tetraphylla*
**6**	*T. turkestanica*	*T. turkestanica*
**8**	*T. korolkowii*	*T. korolkowii*
**9**	*T. dasystemon*	*T. dasystemon*
**10**	*T. fosteriana* (*affinis*)	*T. affinis*
**17**	*T. platystemon*	*T. platystemon*
**22**	*T. urumiensis* (*tarda*)	*T. urumiensis*
**23**	Sp. unknown	*T. brachystemon*
**27**	*T. ostrowskiana*	*T. ostrowskiana*
**35**	*T. greigii*	*T. greigii*
**40**	*T. dasystemon* (*dasystemonoides*)	*T. neustruevae*
**42**	*T. × tschimganica* (*anadroma*)	*T. anadroma*
**43**	*T. zonneveldii*	*T. zonneveldii*

**48**	*T. dubia*	*T. dubia*
**49**	*T. bifloriformis*	*T. bifloriformis*
**50**	*T. kaufmanniana*	*T. kaufmanniana*
**Jac**	*T. jacquesii*	*T. jacquesii*
**Tjk2**	*T. linifolia*	*T. linifolia*
**Tjk4**	*T. subquinquefolia*	*T. subquinquefolia*
**Tjk5**	*T. anisophylla*	*T. anisophylla*
**Tjk7**	*T. vvedenskyi*	*T. vvedenskyi*
**Tjk9**	*T. praestans*	*T. praestans*
**Tjk10**	*T. hissarica*	*T. hissarica*
**Tjk12**	*T. lanata*	*T. lanata*
**Bal1**	*T. albanica*	*T. albanica*
**Bal13**	*T. scardica*	*T. scardica*
**Bal15**	*T. serbica*	*T. serbica*
**Bal19**	*T. sylvestris* (ssp. *sylvestris*)	*T. sylvestris*
**Kyr1**	*T. heterophylla*	*T. heterophylla*
**Kyr5**	*T. kolpakowskiana*	*T. kolpakowskiana*
**Kyr6**	*T. talassica*	*T. talassica*
**Tur1**	*T. koyuncui*	*T. biflora*
**Tur6**	*T. humilis*	*T. humilis*
**Zonn4**	*T. kolbintsevii*	*T. kolbintsevii*
**Zonn8**	*T. ivasczenkoae*	*T. ivasczenkoae*
**Zonn9**	*T. regelii*	*T. regelii*
**Zonn11**	*T. heteropetala*	*T. heteropetala*
**Zonn14**	*T. dianaeverettiae*	*T. dianaeverettiae*
**Got2**	*T. julia*	*T. julia*
**Got6**	*T. uniflora*	*T. uniflora*
**Herb16**	*T. kuschkensis*	*T. kuschkensis*
**Herb20**	*T. armena*	*T. armena*
**MT327741**	*T. iliensis* (*thianschanica*)	*T. iliensis*
**MW077739**	*T. sylvestris* (*patens*)	*T. patens*
**Uzb1**	*T. uzbekistanica*	*T. uzbekistanica*
**Uzb2**	*T. × tschimganica*	*T. × tschimganica*
**Uzb3**	*T. orithyioides*	*T. orithyioides*
**Uzb7**	*T. fosteriana*	*T. fosteriana*
**Uzb10**	*T. ingens*	*T. ingens*
**Uzb15**	*T. lehmanniana*	*T. lehmanniana*
**Uzb17**	*T. scharipovii*	*T. scharipovii*
**Uzb20**	*T. butkovii*	*T. butkovii*

2
