# Supplementary material for: Phylogenomic insights into the genus Tulipa (Liliaceae): Taxonomy, evolution, and biogeography
Source: Am J Bot. 2026 Aug 4;113(8):e70232. doi: 10.1002/ajb2.70232 (PMC13496073; doi:10.1002/ajb2.70232)
Supplement: Supplementary file 9 [file AJB2-113-e70232-s009.pdf]

Appendix S8. All genes annotated in the *Tulipa* plastid assemblies

Groups of gene	Name of gene
**Ribosomal RNAs**	rrn16 (×2), rrn23 (×2), rrn4.5 (×2), rrn5 (×2)
**Transfer RNAs**	trnA-UGC* (×2), trnC-GCA, trnD-GUC, trnE-UUC, trnF-GAA, trnfM-CAU, trnG-GCC*, trnG-UCC, trnH-GUG (×2), trnI-CAU (×2), trnI-GAU* (×2), trnK-UUU*, trnL-CAA (×2), trnL-UAA*, trnL-UAG, trnM-CAU, trnN-GUU (×2), trnP-UGG, trnQ-UUG, trnR-ACG (×2), trnR-UCU, trnS-GCU, trnS-GGA, trnS-UGA, trnT-GGU, trnT-UGU, trnV-GAC (×2), trnV-UAC*, trnW-CCA, trnY-GUA
**Photosystem I**	*PSAA*, *PSAB*, *PSAC*, *PSAI*, *PSAJ*
**Photosystem II**	*PSBA*, *PSBB*, *PSBC*, *PSBD*, *PSBE*, *PSBF*, *PSBH*, *PSBI*, *PSBJ*, *PSBK*, *PSBL*, *PSBM*, *PSBN*, *PSBT*, *PSBZ*
**Cytochrome**	*PETA*, *PETB**, *PETD**, *PETG*, *PETL*, *PETN*
**ATP synthase**	*ATPA*, *ATPB*, *ATPE*, *ATPF**, *ATPH*, *ATPI*
**Rubisco**	*RBCL*
**NADH dehydrogenase**	*NDHA**, *NDHB** (×2), *NDHC*, *NDHD*, *NDHE*, *NDHF*, *NDHG*, *NDHH*, *NDHI*, *NDHJ*, *NDHK*
**ATP-dependent protease subunit P**	*CLPP*†
**Chloroplast envelope membrane protein**	*CEMA*
**Large units**	*RPL2** (×2), *RPL14*, *RPL16**, *RPL20*, *RPL22*, *RPL23* (×2), *RPL32*, *RPL33*, *RPL36*
**Small units**	*RPS2*, *RPS3*, *RPS4*, *RPS7* (×2), *RPS8*, *RPS11*, *RPS12*˜ (×2), *RPS14*, *RPS15*, *RPS16**, *RPS18*, *RPS19* (x2 - one copy is #)
**RNA polymerase**	*RPOA*, *RPOB*, *RPOC1**, *RPOC2*
**Miscellaneous proteins**	*MATK*, *ACCD*, *CCSA*
**Hypothetical proteins & Conserved reading frame**	*YCF1* (x2 - one copy is #), *YCF2* (×2), *YCF3*†, *YCF4*, *YCF15*# (×2), *YCF68*# (×2)

*** A gene containing a single intron**

† A gene containing two introns

# Pseudogenes (genes with internal stop codons and unknown functions)

˜ Truncated gene

(×2) A gene duplicated in the IR regions
