# Supplementary material for: Phylogenomic insights into the genus Tulipa (Liliaceae): Taxonomy, evolution, and biogeography
Source: Am J Bot. 2026 Aug 4;113(8):e70232. doi: 10.1002/ajb2.70232 (PMC13496073; doi:10.1002/ajb2.70232)
Supplement: Supplementary file 10 [file AJB2-113-e70232-s010.pdf]

Appendix S13. Geological time scale of epochs and ages during the existence of the genus *Tulipa*.

Epoch	Age	Began (Mya)
**Holocene**	Meghalayan	0.0042
	Northgrippian	0.0082
	Greenlandian	0.0117
**Pleistocene**	Late Tarantian	0.129
	Chibanian	0.774
	Calabrian	1.8
	Gelasian	2.58
**Pliocene**	Piacenzian	3.6
	Zanclean	5.333
**Miocene**	Messinian	7.246
	Tortonian	11.63
	Serravallian	13.82
	Langhian	15.97
	Burdigalian	20.44
	Aquitanian	23.03
**Oligocene**	Chattian	28.1
	Rupelian	33.9
**Eocene**	Priabonian	37.8
	Bartonian	41.2
	Lutetian	47.8
	Ypresian	56.0
