# Supplementary material for: Phylogenomic insights into the genus Tulipa (Liliaceae): Taxonomy, evolution, and biogeography
Source: Am J Bot. 2026 Aug 4;113(8):e70232. doi: 10.1002/ajb2.70232 (PMC13496073; doi:10.1002/ajb2.70232)
Supplement: Supplementary file 11 [file AJB2-113-e70232-s011.pdf]

Appendix S7. Details of the 245 specimens successfully sequenced for this project. Both accepted taxon name based on Table 1.3 and synonym names given under named as where relevant. Code refers to the name given to material in the lab to link it to a specimen. Country, given as the ISO alpha-3 country code or GO if garden origin, refers to where material was originally sourced. Sources are wild, given as an herbarium acronym, or when from a living collection marked with a * and a given acronym (CUBG – Cambridge University Botanic Garden, RBK – Royal Botanic Gardens Kew, RBE – Royal Botanic Gardens Edinburgh, GBG – Gothenburg Botanical Garden, and PC – private collection). Size refers to the overall plastome assembly length in base pairs, with GC% given and length of large single-copy region (LSC), inverted repeat (IR), and small single-copy region (SSC) also reported in base pairs

**Code**	Accepted Taxon	Named as	Country	Source	Size, bp	GC %	LSC	IR	SSC
**1**	Tulipa biflora	binutans	KGZ	Wild	152016	36.6	82153	26332	17199
**2**	Tulipa lehmanniana	zenaidae	KGZ	Wild	152023	36.6	82192	26334	17163
**3**	Tulipa ferganica	-	KGZ	Wild	152135	36.7	82212	26372	17179
**4**	Tulipa talassica	-	KGZ	Wild	152215	36.6	82372	26344	17155
**5**	Tulipa tetraphylla	-	KGZ	Wild	152072	36.6	82224	26342	17164
**6**	Tulipa turkestanica	-	KGZ	Wild	152040	36.6	82199	26331	17179
**7**	Tulipa korolkowii	rosea	KGZ	Wild	152090	36.6	82256	26339	17156
**8**	Tulipa korolkowii	-	KGZ	Wild	152071	36.6	82236	26339	17157
**9**	Tulipa dasystemon	-	KGZ	Wild	152097	36.6	82240	26336	17185
**10**	Tulipa fosteriana	affinis	KGZ	Wild	152119	36.6	82171	26369	17210
**13**	Tulipa turkestanica	-	KGZ	Wild	152024	36.6	82162	26332	17198
**14**	Tulipa fosteriana	affinis	KGZ	Wild	152152	36.6	82203	26369	17211
**16**	Tulipa ferganica	-	KGZ	Wild	152145	36.7	82225	26372	17176
**17**	Tulipa platystemon	-	KGZ	Wild	152114	36.6	82305	26326	17157
**18**	Tulipa ferganica	-	KGZ	Wild	152140	36.7	82227	26372	17169
**22**	Tulipa urumiensis	tarda	KGZ	Wild	152015	36.6	82138	26335	17207
**23**	Sp. unknown (Chuy)	-	KGZ	Wild	152090	36.6	82281	26326	17157

**27**	Tulipa ostrowskiana	-	KGZ	Wild	152098	36.6	82290	26326	17156
**31**	Tulipa dasystemon	-	KGZ	Wild	152118	36.6	82258	26336	17188
**34**	Tulipa lehmanniana	zenaidae	KGZ	Wild	152018	36.7	82199	26334	17151
**35**	Tulipa greigii	-	KGZ	Wild	152248	36.6	82259	26371	17247
**39**	Tulipa tetraphylla	-	KGZ	Wild	152057	36.6	82209	26342	17164
**40**	Tulipa dasystemon	dasystemonoides	KGZ	Wild	152072	36.6	82161	26346	17219
**41**	Tulipa × tschimganica	anadroma	KGZ	Wild	152458	36.6	82528	26359	17212
**42**	Tulipa × tschimganica	anadroma	KGZ	Wild	152333	36.6	82436	26359	17179
**43**	Tulipa zonneveldii	-	KGZ	Wild	152455	36.6	82529	26359	17208
**44**	Tulipa zonneveldii	-	KGZ	Wild	152354	36.6	82438	26358	17200
**45**	Tulipa ferganica.	pseudoferganica	KGZ	Wild	152156	36.7	82212	26372	17200
**48**	Tulipa dubia	-	KGZ	Wild	152171	36.6	82259	26365	17182
**49**	Tulipa bifloriformis	-	KGZ	Wild	152042	36.6	82171	26330	17211
**50**	Tulipa kaufmanniana	-	KGZ	Wild	152228	36.6	82241	26371	17245
**51**	Sp. unknown (Chatkal)	-	KGZ	Wild	152004	36.6	82164	26331	17178
**52**	Tulipa kaufmanniana	-	KGZ	Wild	152226	36.6	82239	26371	17245
**57**	Tulipa bifloriformis	-	KGZ	Wild	151981	36.6	82112	26328	17213
**58**	Tulipa greigii	-	KGZ	Wild	152239	36.6	82251	26373	17242
**59**	Sp. unknown (Talas)	-	KGZ	Wild	152029	36.6	82208	26330	17161
**60**	Tulipa ostrowskiana	-	KGZ	Wild	152095	36.6	82287	26326	17156
**62**	Tulipa greigii	-	KGZ	Wild	152245	36.6	82257	26371	17246

**Jac**	Tulipa jacquesii	-	KGZ	Wild	151,751	36.6	81893	26337	17184
**A**	Tulipa biflora	-	KAZ	CUBG*	152036	36.6	82174	26332	17198
**B**	Tulipa bifloriformis	-	KAZ	CUBG*	151973	36.6	82134	26331	17177
**C**	Tulipa lehmanniana	-	KAZ	CUBG*	151137	36.7	81340	26353	17091
**D**	Tulipa saxatilis	-	GRC	CUBG*	152057	36.7	82179	26335	17208
**D2**	Tulipa saxatilis	-	GRC	CUBG*	152062	36.6	82184	26334	17210
**E**	Tulipa greigii	-	KAZ	CUBG*	152246	36.6	82244	26373	17256
**E2**	Tulipa greigii	-	UZB	RBK*	152556	36.6	82533	26363	17297
**F**	Tulipa montana	-	IRN	CUBG*	151589	36.7	81599	26391	17208
**G**	Tulipa kaufmanniana	-	KAZ	CUBG*	152243	36.6	82245	26373	17252
**H**	Tulipa tetraphylla	-	KAZ	CUBG*	152067	36.6	82218	26342	17165
**I**	Tulipa turkestanica	-	KAZ	CUBG*	152031	36.6	82195	26330	17176
**J**	Tulipa dasystemon	-	KAZ	CUBG*	152120	36.6	82263	26336	17185
**K**	Tulipa dasystemon	-	KAZ	CUBG*	152094	36.6	82239	26336	17183
**L**	Tulipa vvedenskyi	-	UZB	RBK*	152226	36.6	82241	26372	17241
**L2**	Tulipa vvedenskyi	-	UZB	RBK*	152246	36.6	82260	26371	17244
**M**	Tulipa × tschimganica	-	UZB	RBK*	152255	36.6	82269	26372	17242
**N**	Tulipa ingens	tubergeniana	GO	RBK*	152366	36.6	82421	26357	17231
**O**	Tulipa undulatifolia	-	GO	RBK*	152410	36.6	82417	26363	17267
**P**	Tulipa ostrowskiana	-	KAZ	RBK*	152099	36.6	82291	26326	17156
**Q**	Tulipa biflora	sogdiana	GO	RBK*	152016	36.6	82173	26331	17181
**R**	Tulipa orphanidea	-	TUR	RBK*	151850	36.6	82243	26197	17213
**S**	Tulipa humilis	-	TUR	RBK*	152054	36.6	82174	26333	17214

**T**	Tulipa clusiana (f. cashmeriana)	-	GO	RBK*	151649	36.7	81696	26370	17213
**U**	Tulipa urumiensis	tarda	KAZ	RBK*	152001	36.6	82140	26335	17191
**V**	Tulipa butkovii	-	UZB	RBK*	152242	36.6	82254	26371	17246
**W**	Tulipa lemmersii	-	KAZ	CUBG*	152100	36.6	82299	26334	17133
**X**	Tulipa Duc van Tol	-	GO	CUBG*	151997	36.6	82175	26351	17120
**Kew 1**	Tulipa foliosa	armena var lycica	TUR	RBK*	152429	36.5	82372	26389	17279
**Kew 2**	Tulipa biflora	binutans	KAZ	RBK*	151899	36.6	82150	26332	17085
**Kew 4**	Tulipa carinata	-	UZB	RBK*	152425	36.6	82469	26366	17224
**Kew 5**	Tulipa clusiana	-	IRN	RBK*	151583	36.7	81642	26370	17201
**Kew 6**	Tulipa clusiana	aitchisonii	AFG	RBK*	151535	36.7	81594	26350	17241
**Kew 7**	Tulipa dasystemon	dasystemonoides	UZB	RBK*	152152	36.6	82311	26331	17179
**Kew 8**	Tulipa kaufmanniana	-	KAZ	RBK*	152244	36.6	82258	26371	17244
**Kew 9**	Tulipa montana	chrysantha	IRN	RBK*	151569	36.7	81612	26370	17217
**Kew 10**	Tulipa orithyioides	-	UZB	RBK*	152059	36.6	82203	26332	17192
**Kew 11**	Tulipa orphanidea	hageri	TUR	RBK*	152091	36.6	82210	26333	17215
**Kew 12**	Tulipa orphanidea	theophrastii	GRC	RBK*	151894	36.6	82244	26197	17256
**Kew 13**	Tulipa praestans		TJK	RBK*	152211	36.6	82279	26368	17196
**Kew 14**	Tulipa saxatilis	bakeri	GRC	RBK*	152068	36.6	82194	26333	17208
**Kew 15**	Sp. unknown (Varzob)	-	TJK	RBK*	152171	36.6	82227	26367	17210
**Kew 16**	Tulipa sylvestris	biebersteiniana	IRN	RBK*	151857	36.6	82238	26197	17225
**Kew 17**	Tulipa sylvestris	patens	KAZ	RBK*	151997	36.6	82140	26332	17193
**Kew 18**	Tulipa systola	-	IRN	RBK*	152349	36.6	82339	26371	17268
**Kew 19**	Tulipa × tschimganica	-	UZB	RBK*	152232	36.6	82245	26372	17243
**Kew 20**	Tulipa turkestanica	-	KAZ	RBK*	151886	36.6	82237	26197	17255

**Kew 21**	Tulipa undulatifolia	micheliana	IRN	RBK*	152308	36.6	82341	26371	17225
**Kew 22**	Tulipa borszczowii	-	KAZ	RBK*	151059	36.7	81321	26353	17032
**Kew 23**	Tulipa hungarica	-	BGR	RBK*	152057	36.6	82228	26351	17127
**Kew 24**	Tulipa lemmersii	-	KAZ	RBK*	152088	36.6	82287	26334	17133
**Kew 25**	Tulipa hungarica	rhodopea	BGR	RBK*	152090	36.6	82257	26352	17129
**Kew 26**	Tulipa talassica	-	KGZ	RBK*	152078	36.6	82272	26334	17138
**Kew 27**	Tulipa agenensis	-	ISR	RBK*	152281	36.6	82338	26383	17177
**Kew 28**	Tulipa alberti	-	KAZ	RBK*	152374	36.6	82377	26372	17253
**Kew 29**	Tulipa altaica	-	KAZ	RBK*	151633	36.7	81698	26352	17231
**Kew 31**	Tulipa cinnabarina	-	TUR	RBK*	151892	36.6	82183	26386	17369
**Kew 32**	Tulipa cretica	-	GRC	RBK*	152028	36.6	82152	26334	17208
**Kew 33**	Tulipa systola	florenskyi	ARM	RBK*	152675	36.5	82620	26385	17285
**Kew 34**	Tulipa hoogiana	-	IRN	RBK*	152313	36.6	82346	26371	17225
**Kew 35**	Tulipa hoogiana	-	IRN	RBK*	152309	36.6	82341	26371	17226
**Kew 36**	Tulipa ingens	-	TUR	RBK*	152329	36.6	82344	26371	17243
**Kew 37**	Tulipa jacquesii	-	KGZ	RBK*	152145	36.6	82254	26369	17153
**Kew 38**	Tulipa kolpakowskiana	-	KAZ	RBK*	152149	36.7	82228	26373	17175
**Kew 39**	Tulipa undulatifolia	micheliana	IRN	RBK*	152338	36.6	82395	26373	17197
**Kew 40**	Tulipa montana	-	IRN	RBK*	151583	36.7	81594	26391	17207
**Kew 42**	Tulipa suaveolens	schrenkii	KAZ	RBK*	152371	36.6	82379	26373	17246
**Kew 43**	Tulipa suaveolens	schrenkii	RUS	RBK*	152090	36.6	82261	26352	17125
**Kew 44**	Tulipa sosnowskyi	-	ARM	RBK*	152674	36.5	82620	26385	17284
**Kew 45**	Tulipa systola	stapfi	IRN	RBK*	152304	36.6	82336	26371	17226
**Kew 46**	Tulipa sylvestris	australis talijevii	KAZ	RBK*	152041	36.6	82180	26333	17195
**Kew 47**	Tulipa ulophylla	-	IRN	RBK*	152308	36.6	82340	26371	17226

**Kew 48**	Tulipa uniflora	-	RUS	RBK*	152120	36.6	82266	26355	17144
**Kew 49**	Tulipa dasystemon	neustruevae	UZB	RBK*	152162	36.6	82264	26343	17212
**Herb 1**	Tulipa albanica	-	ALB	C	152162	36.6	82252	26350	17210
**Edi1**	Tulipa agenensis	-	GO	RBE*	152391	36.6	82353	26370	17298
**Edi2**	Tulipa cretica	-	GO	RBE*	152073	36.6	82195	26334	17210
**Edi3**	Tulipa gesneriana	-	RUS	RBE*	152127	36.6	82217	26354	17202
**Edi4**	Tulipa lanata	-	TJK	RBE*	152416	36.6	82388	26357	17314
**Edi5**	Tulipa praestans	-	TJK	RBE*	152231	36.6	82241	26372	17246
**Edi6**	Tulipa heterophylla	-	KGZ	RBE*	152134	36.6	82276	26336	17186
**Edi7**	Tulipa sprengeri	-	GO	RBE*	152362	36.5	82474	26354	17180
**Tjk1**	Tulipa praestans	-	TJK	Wild	152216	36.6	82283	26368	17197
**Tjk2**	Tulipa linifolia	-	TJK	Wild	151477	36.7	81575	26350	17202
**Tjk3**	Tulipa linifolia	-	TJK	Wild	151477	36.7	81575	26350	17202
**Tjk4**	Tulipa subquinquefolia	-	TJK	Wild	152412	36.6	82384	26357	17314
**Tjk5**	Tulipa anisophylla	-	TJK	Wild	152094	36.6	82202	26357	17178
**Tjk6**	Tulipa praestans	subpraestans	TJK	Wild	152097	36.6	82179	26361	17196
**Tjk7**	Tulipa vvedenskyi	-	TJK	Wild	152552	36.6	82537	26379	17257
**Tjk8**	Tulipa ingens	-	TJK	Wild	152427	36.6	82398	26364	17301
**Tjk9**	Tulipa praestans	-	TJK	Wild	152166	36.6	82223	26367	17209
**Tjk10**	Tulipa hissarica	-	TJK	Wild	152450	36.6	82463	26359	17269
**Tjk11**	Tulipa anisophylla	korshinskyi	TJK	Wild	152090	36.6	82207	26357	17169
**Herb 2**	Tulipa scardica	-	MKD	B	151658	36.6	81838	26349	17137
**Bal1**	Tulipa albanica	-	ALB	Wild	152167	36.6	82257	26350	17210
**Bal2**	Tulipa albanica	-	ALB	Wild	152252	36.6	82435	26258	17210
**Bal3**	Tulipa albanica	-	ALB	Wild	152170	36.6	82259	26350	17211
**Bal4**	Tulipa kosovarica	-	XKX	Wild	152410	36.6	82248	26350	17209

**Bal5**	Tulipa kosovarica	-	XKX	Wild	152158	36.6	82247	26350	17211
**Bal6**	Tulipa kosovarica	-	XKX	Wild	152154	36.6	82245	26350	17209
**Bal7**	Tulipa kosovarica	-	XKX	Wild	152151	36.6	82242	26350	17209
**Bal8**	Tulipa sp. nov.	-	XKX	Wild	152231	36.6	82263	26349	17270
**Bal9**	Tulipa luanica	-	XKX	Wild	152147	36.6	82242	26349	17207
**Bal10**	Tulipa luanica	-	XKX	Wild	152147	36.6	82242	26349	17207
**Bal11**	Tulipa luanica	-	XKX	Wild	152140	36.6	82236	26349	17206
**Bal12**	Tulipa luanica	-	XKX	Wild	152156	36.6	82247	26350	17209
**Bal13**	Tulipa scardica	-	XKX	Wild	152101	36.6	82260	26350	17141
**Bal14**	Tulipa scardica	-	XKX	Wild	152100	36.6	82259	26350	17141
**Bal15**	Tulipa serbica	-	XKX	Wild	152149	36.6	82239	26350	17210
**Bal16**	Tulipa serbica	-	XKX	Wild	152149	36.6	82239	26350	17210
**Bal17**	Tulipa sylvestris	Subsp. sylvestris	XKX	Wild	151924	36.6	82207	26230	17257
**Bal18**	Tulipa sylvestris	Subsp. australis	XKX	Wild	151887	36.6	82235	26197	17258
**Bal19**	Tulipa sylvestris	Subsp. sylvestris	XKX	Wild	151879	36.6	82228	26197	17257
**Kyr1**	Tulipa heterophylla	-	KGZ	Wild	151833	36.7	81822	26419	17173
**Kyr2**	Tulipa heterophylla	-	KGZ	Wild	151852	36.7	81836	26418	17180
**Kyr3**	Tulipa heterophylla	-	KGZ	Wild	151836	36.7	81852	26404	17176
**Kyr4**	Tulipa heterophylla	-	KGZ	Wild	151867	36.7	81832	26429	17177
**Kyr5**	Tulipa kolpakowskiana	-	KGZ	Wild	152101	36.6	82293	26326	17156
**Kyr6**	Tulipa talassica	-	KGZ	Wild	152078	36.6	82273	26334	17137
**Kyr7**	Tulipa jacquesii	-	KGZ	Wild	151751	36.6	81914	26337	17163
**Tur1**	Tulipa koyuncui	-	TUR	Wild	152040	36.6	82194	26333	17180
**Tur2**	Tulipa biflora	-	TUR	Wild	152039	36.6	82194	26333	17179

**Tur3**	Tulipa biflora	-	TUR	Wild	152047	36.6	82201	26333	17180
**Tur4**	Sp. unknown	-	TUR	Wild	152039	36.6	82194	26333	17179
**Tur5**	Tulipa koyuncui	-	TUR	Wild	152049	36.6	82202	26333	17181
**Tur6**	Tulipa humilis	-	TUR	Wild	151781	36.7	82170	26199	17213
**Tur7**	Tulipa biflora	-	TUR	Wild	152039	36.6	82194	26333	17179
**Tur8**	Tulipa biflora	-	TUR	Wild	152039	36.6	82194	26333	17179
**Tur9**	Sp. unknown	-	TUR	Wild	152041	36.6	82195	26333	17180
**Tur10**	Sp. unknown	-	TUR	Wild	152039	36.6	82194	26333	17179
**Tur11**	Tulipa koyuncui	-	TUR	Wild	152049	36.6	82202	26333	17181
**Tur12**	Tulipa koyuncui	-	TUR	Wild	152039	36.6	82194	26333	17179
**Zonn3**	Tulipa lemmersii	-	KAZ	PC*	152100	36.6	82299	26334	17133
**Zonn4**	Tulipa kolbintsevii	-	KAZ	PC*	152067	36.6	82221	26323	17200
**Zonn5**	Tulipa zonneveldii	-	KGZ	PC*	152389	36.6	82456	26358	17217
**Zonn6**	Tulipa jacquesii	-	KGZ	PC*	151737	36.6	81898	26337	17165
**Zonn7**	Tulipa annae	-	KAZ	PC*	151718	36.6	81799	26354	17211
**Zonn8**	Tulipa ivasczenkoae	-	KAZ	PC*	152454	36.6	82428	26363	17300
**Zonn9**	Tulipa regelii	-	KAZ	PC*	152114	36.6	82202	26337	17238
**Zonn10**	Tulipa uzbekistanica	-	UZB	PC*	152387	36.6	82342	26362	17321
**Zonn11**	Tulipa heteropetala	-	KAZ	PC*	152361	36.6	82405	26359	17238
**Zonn12**	Tulipa alberti	-	KAZ	PC*	152379	36.6	82378	26372	17257
**Zonn13**	Tulipa alberti	-	KAZ	PC*	152391	36.6	82384	26367	17273
**Zonn14**	Tulipa dianaeverettiae	-	KAZ	PC*	152005	36.6	82149	26332	17192
**Chr1**	Tulipa heweri	-	GO	PC*	152291	36.6	82358	26363	17207
**Herb4**	Tulipa julia	-	TUR	E	152526	36.6	82555	26371	17229

**Herb 5**	Tulipa kuschkensis	-	IRN	E	151505	36.7	81613	26343	17206
**Herb 6**	Tulipa lehmanniana	-	AFG	E	151121	36.7	81328	26351	17091
**Herb 7**	Tulipa gesneriana	-	IRN	E	152428	36.6	82387	26393	17255
**Herb 8**	Tulipa banuensis	-	AFG	E	152284	36.6	82356	26363	17202
**Herb 9**	Tulipa lehmanniana	-	AFG	E	151139	36.7	81336	26353	17097
**Herb 10**	Tulipa sosnowskyi	-	ARM	E	152673	36.5	82619	26385	17284
**Herb 11**	Tulipa banuensis	-	AFG	E	152285	36.6	82358	26363	17201
**Herb 14**	Tulipa harazensis	-	IRN	E	152,350	36.6	82340	26371	17268
**Herb 15**	Tulipa hoogiana	-	IRN	E	152,283	36.6	82315	26371	17226
**Herb 16**	Tulipa kuschkensis	-	AFG	E	152419	36.6	82412	26357	17293
**Herb 17**	Tulipa agenensis	-	TUR	E	152200	36.6	82256	26351	17242
**Herb 18**	Tulipa armena	-	TUR	E	152437	36.6	82503	26373	17188
**Herb 19**	Tulipa sosnowskyi	-	ARM/AZE	E	152674	36.5	82620	26385	17284
**Herb 20**	Tulipa armena	-	TUR	E	152554	36.6	82583	26371	17229
**Got2**	Tulipa julia		TUR	GBG*	152503	36.6	82568	26373	17189
**Got3**	Tulipa suaveolens	schrenkii	RUS	GBG*	152144	36.6	82305	26353	17133
**Got4**	Tulipa armena	-	TUR	GBG*	152323	36.6	82429	26343	17208
**Got5**	Tulipa altaica	-	KAZ	GBG*	151560	36.7	81647	26298	17317
**Got6**	Tulipa uniflora	-	RUS	GBG*	152297	36.5	82349	26356	17236
**Got7**	Tulipa regelii	-	KAZ	GBG*	152105	36.6	82195	26337	17236
**Got9**	Tulipa heteropetala	-	KAZ	GBG*	152423	36.6	82469	26359	17236
**Got10**	Tulipa heteropetala	-	KAZ	GBG*	152272	36.6	82316	26359	17238
**Got11**	Tulipa regelii	-	KAZ	GBG*	152117	36.6	82206	26337	17237

**Uzb1**	Tulipa uzbekistanica	-	UZB	Wild	152359	36.6	82334	26362	17301
**Uzb2**	Tulipa × tschimganica	-	UZB	Wild	152166	36.6	82247	26372	17175
**Uzb3**	Tulipa orithyioides	-	UZB	Wild	152067	36.6	82231	26332	17172
**Uzb4**	Tulipa greigii	-	UZB	Wild	152225	36.6	82239	26371	17244
**Uzb5**	Tulipa vvedenskyi	-	UZB	Wild	152198	36.6	82210	26372	17244
**Uzb6**	Tulipa carinata	-	UZB	Wild	152453	36.6	82461	26363	17266
**Uzb7**	Tulipa fosteriana	-	UZB	Wild	152457	36.6	82432	26363	17299
**Uzb8**	Tulipa korolkowii	-	UZB	Wild	152071	36.6	82250	26339	17143
**Uzb9**	Tulipa lanata	-	UZB	Wild	152318	36.6	82374	26357	17230
**Uzb10**	Tulipa ingens	-	UZB	Wild	152465	36.6	82468	26363	17271
**Uzb11**	Tulipa biflora	-	UZB	Wild	151982	36.6	82144	26334	17170
**Uzb12**	Tulipa kaufmanniana	-	UZB	Wild	152167	36.6	82135	26373	17286
**Uzb13**	Tulipa dubia	-	UZB	Wild	152290	36.6	82305	26371	17243
**Uzb14**	Tulipa turkestanica	-	UZB	Wild	152027	36.6	82186	26330	17181
**Uzb15**	Tulipa lehmanniana	-	UZB	Wild	151169	36.7	81346	26354	17115
**Uzb16**	Tulipa talassica	-	UZB	Wild	152067	36.6	82246	26339	17143
**Uzb17**	Tulipa scharipovii	-	UZB	Wild	151989	36.7	82110	26362	17155
**Uzb18**	Tulipa intermedia	-	UZB	Wild	152141	36.6	82275	26339	17188
**Uzb19**	Tulipa ferganica	-	UZB	Wild	152123	36.7	82202	26372	17177
**Uzb20**	Tulipa butkovii	-	UZB	Wild	152167	36.6	82135	26373	17286
**Uzb21**	Tulipa bifloriformis	-	UZB	Wild	152038	36.6	82171	26332	17203
**Got1**	Tulipa cinnabarina	-	TUR	GBG*	151112	36.6	82232	26333	17214
**Got8**	Tulipa kolbintsevii	-	KAZ	GBG*	152060	36.7	82216	26323	17198
**Tjk12**	Tulipa lanata	-	TJK	Wild	152420	36.6	82417	26357	17289

**Tjk13**	Tulipa korolkowii	nitida	TJK	Wild	152002	36.6	82183	26339	17141
**Tjk14**	Tulipa linifolia	maximowiczii	TJK	Wild	152020	36.6	82099	26373	17175
**Tjk15**	Tulipa korolkowii	rosea	TJK	Wild	152061	36.6	82225	26339	17158
**Kyr8**	Tulipa platystemon	-	KGZ	Wild	152118	36.6	82309	26326	17157
**Kyr9**	Tulipa dubia	-	KGZ	Wild	152281	36.6	82370	26365	17181
**Kyr10**	Tulipa korolkowii	-	KGZ	Wild	152137	36.6	82276	26339	17183
**Kyr11**	Tulipa tetraphylla	brachystemon	KGZ	Wild	152101	36.6	82292	26326	17157
**Kyr12**	Tulipa lehmanniana	zenaidae	KGZ	Wild	151974	36.7	82165	26326	17157
**Kyr13**	Tulipa talassica	-	KGZ	Wild	152076	36.6	82270	26334	17138
**Kyr14**	Tulipa talassica	-	KGZ	Wild	152239	36.6	82376	26343	17177
**Kyr15**	Sp. unknown	-	KGZ	Wild	152001	36.6	82161	26331	17178
**Kyr16**	Tulipa dasystemon	-	KGZ	Wild	152048	36.6	82191	26336	17185
**Kyr17**	Tulipa dasystemon	dasystemonoides	KGZ	Wild	152068	36.6	82158	26346	17218
**Kyr18**	Sp. unknown	-	KGZ	Wild	152045	36.6	82175	26331	17208
