# Supplementary material for: Phylogenomic insights into the genus Tulipa (Liliaceae): Taxonomy, evolution, and biogeography
Source: Am J Bot. 2026 Aug 4;113(8):e70232. doi: 10.1002/ajb2.70232 (PMC13496073; doi:10.1002/ajb2.70232)

Appendix S9. Phylogenetic relationships in the tribe Tulipeae inferred from plastome data. (a) Genus-level phylogram. Triangles represent groups containing multiple tips (not scaled to reflect the number of taxa). The tree is rooted with Gagea, and nonparametric bootstrap support values are indicated for each branch. (b) Species-level phylogeny inferred using a maximum likelihood approach. Bootstrap values are shown for all branches. Branches are color-coded to reflect taxonomic or biological insights, including reinstated species, synonymized taxa, species complexes, taxa with high inferred intraspecific variation, and species with uncertain identification.

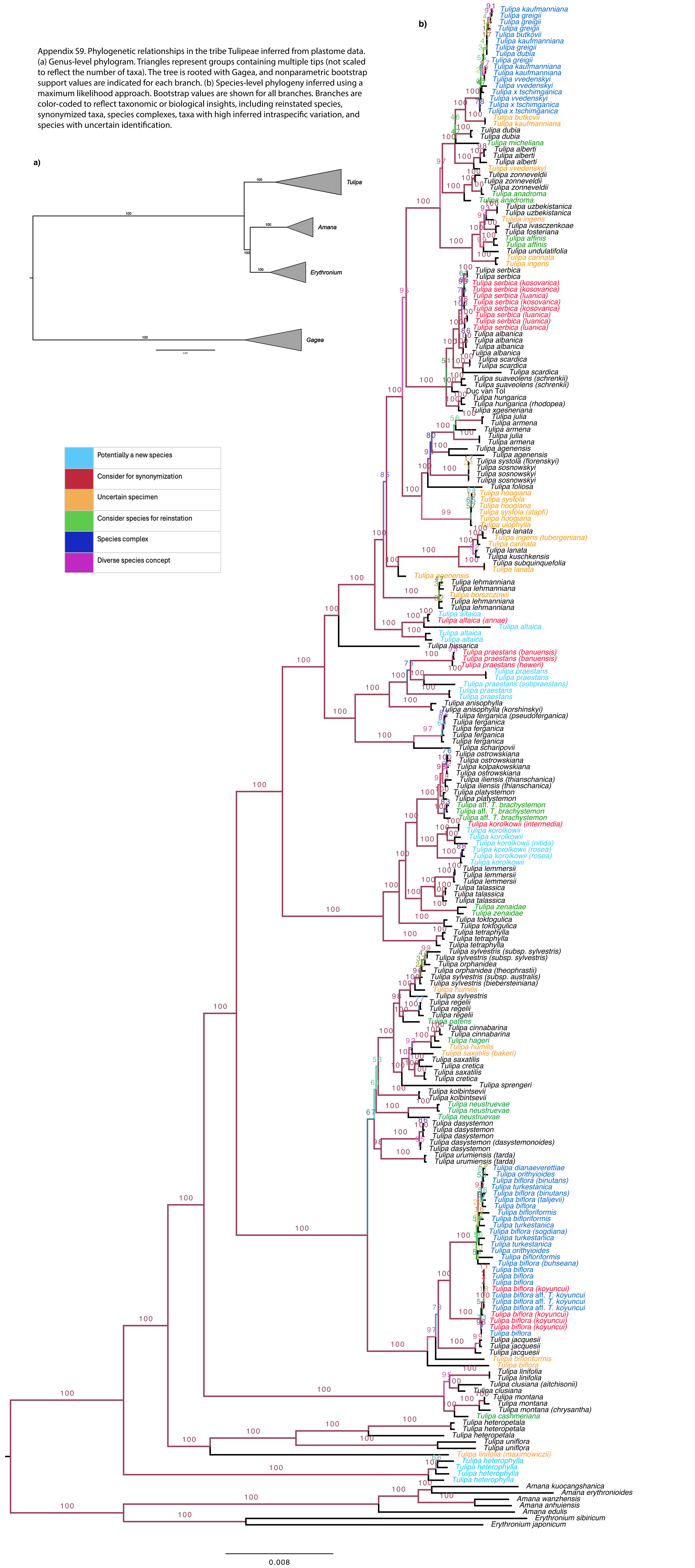

Supplement: Supplementary file 12 [file AJB2-113-e70232-s012.pdf]
