# Supplementary material for: Phylogenomic insights into the genus Tulipa (Liliaceae): Taxonomy, evolution, and biogeography
Source: Am J Bot. 2026 Aug 4;113(8):e70232. doi: 10.1002/ajb2.70232 (PMC13496073; doi:10.1002/ajb2.70232)

Appendix S12. Dated tree and lineage-through-time analyses. (a) Lineage-through-time plot for Tulipa, with the phylogeny overlaid and time measured in millions of years from the origin of the genus. (b) Gamma statistic calculated across 10 sections of the Tulipa genus tree, which is shown in the background for context. (c) Results of MCCR test for Tulipa. The observed gamma value is highlighted. (d) Dated species tree, including Tulipa cv. Duc van Tol, with nodes indicating the estimated divergence times of clades in millions of years ago (Mya).

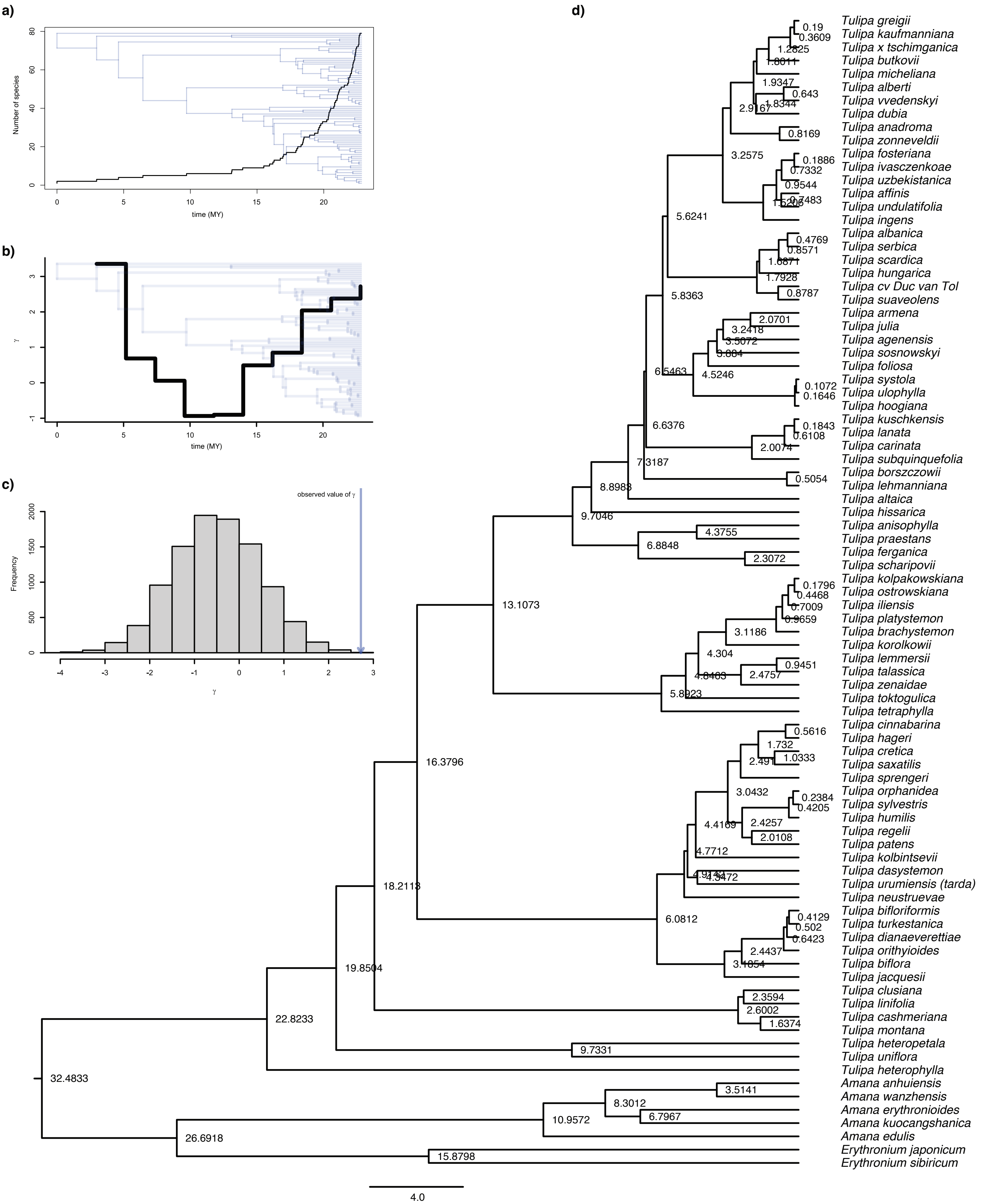

Supplement: Supplementary file 13 [file AJB2-113-e70232-s013.pdf]
